# Separation of 2,3-Butanediol from Fermentation Broth via Cyclic and Simulated Moving Bed Adsorption Over Nano-MFI Zeolites

**DOI:** 10.1021/acssuschemeng.4c04121

**Published:** 2024-09-12

**Authors:** Jianpei Lao, Qiang Fu, Marco Avendano, Jason A. Bentley, Yadong Chiang, Matthew J. Realff, Sankar Nair

**Affiliations:** School of Chemical & Biomolecular Engineering, Georgia Institute of Technology, 311 Ferst Drive NW, Atlanta, Georgia 30332-0100, United States

**Keywords:** butanediol, adsorption, zeolites, biofuel, fermentation, simulated moving bed

## Abstract

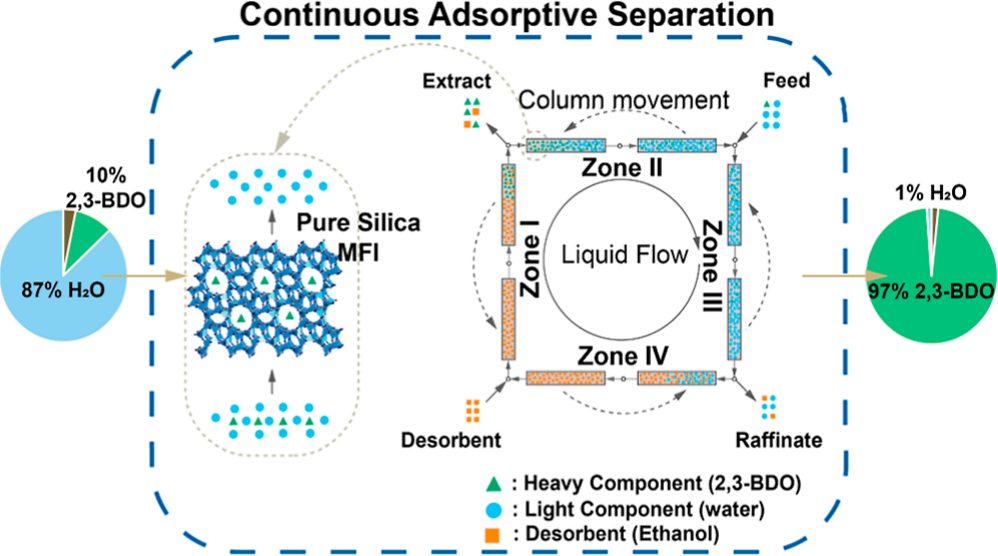

The biomass-based platform molecule 2,3-butanediol (2,3-BDO) has a wide range of applications in production of sustainable fuels, chemicals, synthetic rubber, and others. However, the selective separation of 2,3-BDO from multicomponent fermentation broths presents challenges due to its low concentration, high solubility in water, high boiling point, and presence of many other species. Here, we demonstrate remarkably selective enrichment and recovery of 2,3-BDO from a corn stover hydrolysate fermentation broth by a pure-silica nano-MFI-type zeolite adsorbent. By means of cyclic and simulated moving bed adsorption processes, we obtained concentrated aqueous 2,3-BDO streams from the fermentation process stream with ∼93% purity and 3-fold enrichment, and >98% purity and 8-fold enrichment, respectively. These findings provide strong support for large-scale adsorptive separation for biobased 2,3-BDO production.

## Abstract 2

Sustainable large-scale continuous adsorptive separation of 2,3-BDO from fermentation broth with a nano-MFI zeolite adsorbent increases 2,3-BDO purity from 10 to 97 wt %.

## Introduction

The chemical 2,3-butanediol (2,3-BDO) is an important building block and precursor that has wide applications such as the production of sustainable fuels, synthetic rubber, added-value chemicals, additives, and polymers.^1−5^ The petroleum-based route to 2,3-BDO is via chlorohydrination and hydrolysis of a C_4_ mixture obtained from cracking gases.^6^ More recently, biomass-derived 2,3-BDO production has gained great interest for the production of biojet fuels, via catalytic dehydration to C_4_ olefins followed by oligomerization reactions. The current dramatical increase of concerns on climate change and instability of fossil fuel prices have motivated the shift of 2,3-BDO production from petroleum-based toward biology-based routes. In most biological routes, 2,3-BDO is produced by mixed-acid fermentation with bacterial cells.^1^ The process results in the release of acidic compounds, and the butanediol cycle is then initiated to prevent excessive acidification.^7^*Zymomonas mobilis* is well-known for its high specific glucose uptake rate and rapid catabolism. The anaerobic production of 2,3 BDO in *Z. mobilis* from C_6_/C_5_ sugar streams derived from the deacetylation and mechanical refining process has been demonstrated.^8^ While the resulting concentration of 2,3-BDO from microbial production routes, such as those using *Klebsiella pneumoniae*, can be as high as ∼150 g/L,^9^ the recovery of 2,3-BDO is complicated by the presence of unreacted sugar feedstock, solid/dissolved debris, and fermentation byproducts.^1^ The separation of 2,3-BDO and water is additionally difficult due to its high boiling point (180–184 °C) and affinity for water. In the upgrading path toward biojet fuels, regulation of water content is especially of importance to enhance the conversions and selectivities toward olefins.^10^ The broth characteristics lead to uneconomical results when conventional separations such as distillation are employed.^11,12^

Several alternative separation technologies have been proposed, including liquid–liquid extraction,^13−15^ membrane distillation or pervaporation,^15^ and reactive extraction.^16^ For example, Harvianto et al.^14^ used oleyl alcohol as an extraction solvent, along with vacuum distillation for solvent recovery. This process was simulated to achieve 90% recovery of 2,3-BDO, albeit with high purity (>99%). Additionally, the downstream vacuum distillation uses significant energy for separation of high-boiling oleyl alcohol and 2,3-BDO, albeit less than a conventional direct distillation process. In another example, Shao et al. reported an integrated pervaporation membrane-solvent extraction process, using a polydimethylsiloxane (PDMS) membrane and 1-butanol solvent.^15^ It was shown that pervaporation can significantly reduce the energy input, but the membrane would require a much higher selectivity and resistance to fouling. In general, 2,3-BDO exhibits relatively high hydrophilicity and is difficult to develop scalable membranes that can preferentially permeate 2,3-BDO over water and other species.^17^ Li et al.^21^ demonstrated a reactive extraction process using propionaldehyde as a selective reactant for 2,3-BDO. A recent proposed adaptation^22^ involves the use of butyraldehyde in an acidic environment for extracting 2,3-butanediol from fermentation broth, achieving 99% purity and a 90% recovery. Nevertheless, the method’s scalability is challenged by the suboptimal recovery, the difficulty of recycling the acid catalysts, and equipment corrosion.

**Figure 1:**
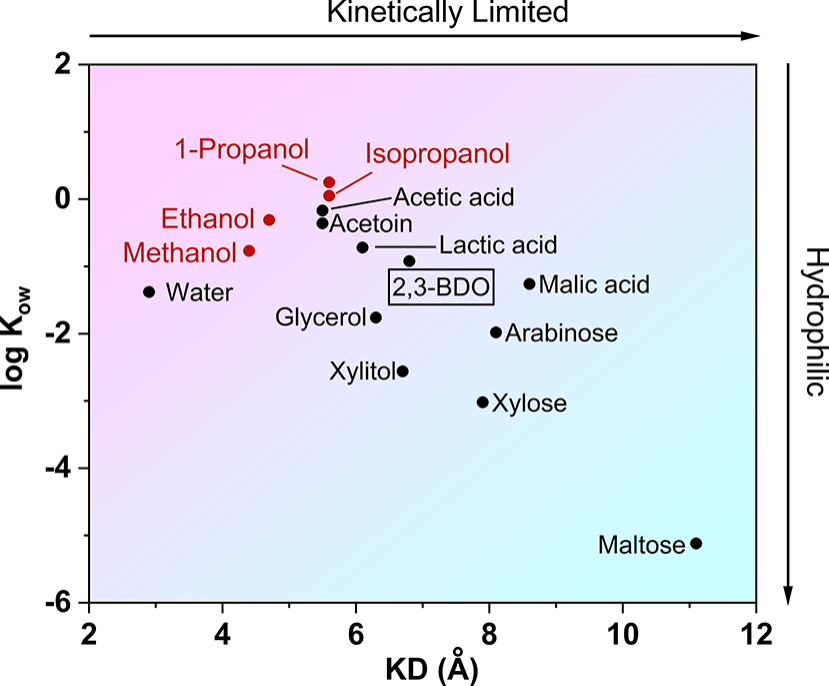
Property map of relevant molecules in the fermentation broth. Tabulated data can be found in Table S1.

Selective adsorption has been proposed in other fermentation systems.^18−22^ Recovery of the adsorbed product is achieved either by temperature cycling (if the product is volatile enough) or more commonly by a liquid desorbent that is then recycled by a distillation column. Depending on the desorbent-to-feed ratio and the ease of desorbent recycling, adsorption processes can lower the costs of biorefinery separations.^22−24^ The hydrophobicity of fermentation-related molecules is depicted using octanol–water partition coefficients (*K*_ow_) and the effective molecular size for diffusion is represented by the kinetic diameter (KD), as shown in Figure 1. The *K*_ow_ is strongly related to the number of hydroxyl groups. Polyols and sugars form a group with larger KD and low hydrophobicity, whereas 2,3-BDO, acetoin, ethanol, and organic acids form another group with higher hydrophobicity and smaller KD. We hypothesized that hydrophobic nanoporous materials with appropriate pore sizes^26−29^ would adsorb 2,3-BDO, whereas sugars and polyols would be rejected. In a recent work,^29^ we showed that nanoporous metal–organic frameworks (MOFs)—specifically zeolitic imidazolate frameworks—could adsorb 2,3-BDO from fermentation broth with high selectivity over the other components including organics, inorganics, and water. Two adsorbents (ZIF-8 and ZIF-71) with small pore sizes (<0.5 nm) suitable for strong adsorption of aliphatic alcohols were investigated. ZIF-8 showed excellent separation performance initially, but displayed structural and separation performance degradation over time in the presence of the desorbent (ethanol). Due to the breakage of the metal–organic linker coordination bond, ZIF-8 lost its framework integrity and lead to a drastic decrease of 2,3-BDO uptake (from 83.4 to 47.0 g/kg adsorbent) and 2,3-BDO/water selectivity (from 76 to 2). While ZIF-71 exhibited a combination of good separation performance, stability in ethanol, and the potential for controllable tuning for separation enhancement, the breakthrough behavior of 2,3-BDO indicated the presence of significant mass transfer limitations owing to the small pore size, even with small (<500 nm) primary particle sizes. Additionally, the synthesis of ZIF-71 involves the use of expensive organic linker, making the scale-up of the separation process less economically attractive.

**Figure 2:**
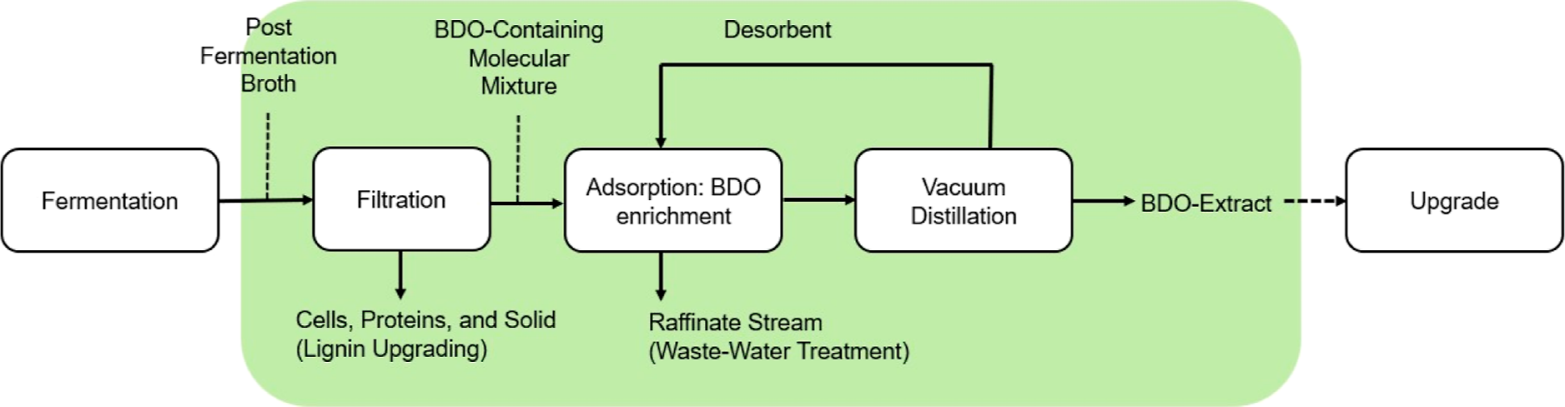
Process flow diagram for the enrichment of BDO from a fermentation broth. The green box depicts the separation processes developed in this work.

To overcome these issues, here we hypothesize that a medium-pore (∼0.6 nm), hydrophobic, and more chemically robust material, such as a zeolite, could provide good 2,3-BDO adsorption selectivity, mass transfer (diffusion) characteristics, as well as excellent chemical stability in the fermentation broth and desorbent. We chose MFI type zeolites^30^ as a potentially desirable adsorbent. MFI is amenable to bulk synthesis, and its hydrophobicity can be controlled by adjusting the Si/Al ratio, for which a pure-SiO_2_ (hydrophobic) MFI would be most suitable in this case. Furthermore, to provide favorable diffusion characteristics,^31^ we synthesized nanosize (<300 nm) MFI materials for fabrication of adsorbent pellets. We measure in detail the adsorption and separation behavior of the MFI adsorbent in the fermentation broth and find excellent separation characteristics and long-term stability. We then develop and experimentally demonstrate the recovery and purification of 2,3-BDO by a model-guided simulated moving bed (SMB) process that depicts the potential for industrial applications. Figure 2 shows a schematic diagram of the overall process with the 2,3-BDO separation process marked by the green box.

## Materials and Methods

### Materials

Chemicals used in the synthesis of pure silica MFI (named GT-MFI in this work), such as tetraethylorthosilicate (TEOS), and 1 M tetrapropylammonium hydroxide (TPAOH) solution in water, were purchased from Sigma-Aldrich. An industrial high silica ZSM-5 MFI (P-1500s, SiO_2_/Al_2_O_3_ > 1500) was purchased from ACS materials, which is named as cMFI in this work. Chemicals used to prepare model broth such as 2,3-butanediol (2,3-BDO mixture of racemic and meso forms), d(+)-glucose, d(+)-xylose, arabinose, malic acid, and xylitol were purchased from Acros Organics. Lactic acid, acetic acid, glycerol, and ethanol were purchased from Fisher Chemical. Maltose was purchased from Fisher BioReagents. The fermentation broth was produced from corn stover hydrolysate according to a published method using the *Z. mobilis* strain^10^ at the National Renewable Energy Laboratory and was received after removing cells and insoluble solids with 0.22 μm PES membrane.

### Pure-Silica MFI Synthesis

The synthesis recipe was adapted from the work by Kasap et al.^25^ 15 mL of 1 M TPAOH solution was added to 12.05 g of DI water while stirring in a 60 mL polypropylene bottle. This was followed by dropwise addition of 12.5 g of TEOS over the span of a few minutes while stirring at 600 rpm to obtain a gel composition of 1 TPAOH:4 TEOS:90 H_2_O. The cap for the polypropylene bottle was closed, and the resultant solution was aged at room temperature for 6 h while stirring at 600 rpm. The gel was then transferred into a 40 mL Teflon-lined autoclave and placed into an oven preheated at 125 °C. The hydrothermal treatment was carried out for 24 h under static conditions in the oven. The oven was then allowed to cool down on its own, and the autoclave was then taken out to recover the MFI crystals. The MFI crystals were recovered by centrifugation and washing with DI water 3 times at 8500 rpm for 15 min each. The solid product was then dried in an oven at 75 °C for 6 h. This was followed by calcination at 550 °C for 6 h with a ramp rate of 2 °C/min (with both heating and cooling) to remove the organic template (TPAOH) and activate the zeolite.

### Materials Characterization

Activated materials were characterized by powder X-ray diffraction (PXRD), nitrogen physisorption, and scanning electron microscope (SEM). PXRD measurements were performed on an X’Pert Pro PANalytical X-ray diffractometer in reflection (Bragg–Brentano) geometry operating with a Cu anode at 45 kV and 40 mA. PXRD patterns were collected with a step size of 0.017° 2θ and scan time of 10 s/step. Surface area and pore volume/size analyses were estimated using nitrogen physisorption isotherms collected at 77 K using a BET surface area analyzer (BELSORP-max, Microtrac). SEM images were taken on a Hitachi SU8010.

#### Pelletization and Packed Column Preparation

The activated materials were loaded into a pellet press die set. The adsorbent particles were prepared without a binder. The pelletization condition was 1000 psi for 60 s. The pellets were ground into small particles, and they were sieved between 425 and 600 μm. From our experience, a pressure higher than 1000 psi may lead to a significant loss of the pore volume. No breakdown of adsorbent was observed through the entire work-frame. The particles were then filled into stainless-steel columns (i.e., 0.94 mm ID × 200 mm L). Both ends of the column were fitted with frits to prevent the loss of adsorbent particles. For enhancing the mechanical strength of the adsorbent, Na_2_SiO_3_ as a binder was incorporated into the MFI pellets.^32^ For example, 13.4 g of as-made pellets were added into binder solutions with a concentration of 5.6 mg/L Na_2_SiO_3_ for 40 min while shaking at 100 rpm (New Brunswick Innova 2000) for good absorption of the binder material. Then, the solution was removed, and the pellets were dried at 70 °C overnight. Finally, the pellets were calcined before being used for column packing.

#### Pretreatment of Fermentation Broth

Nanofiltration (NF) of the broth was carried out in a Sterlitech high-pressure dead-end stirred cell (HP4750X). Ultrahigh purity nitrogen gas supplied the driving pressure with a transmembrane pressure (TMP) of 50 bar, which was monitored by a pressure gauge. The NP010 membranes (Microdyn Nadir, molecular weight cutoff ∼1 kDa) were installed at the bottom of the stirred cell and were supported by a stainless-steel mesh. The fermentation broth was added into the feed chamber, and the whole system was heated with heating tape and kept at 50 °C and monitored by a thermocouple. The NF permeate was collected until an ∼50% volume reduction was reached. Our initial test showed that neutral pH can facilitate the dissociation of the organic acids and reduce their uptake on MFI adsorbents. Therefore, the prefiltered broth (pH ≈ 5.6) is neutralized to pH ≈ 7 with 5 M sodium hydroxide solution before adsorption.

#### Model-Guided SMB Scale-Up Approach

**Figure 3:**
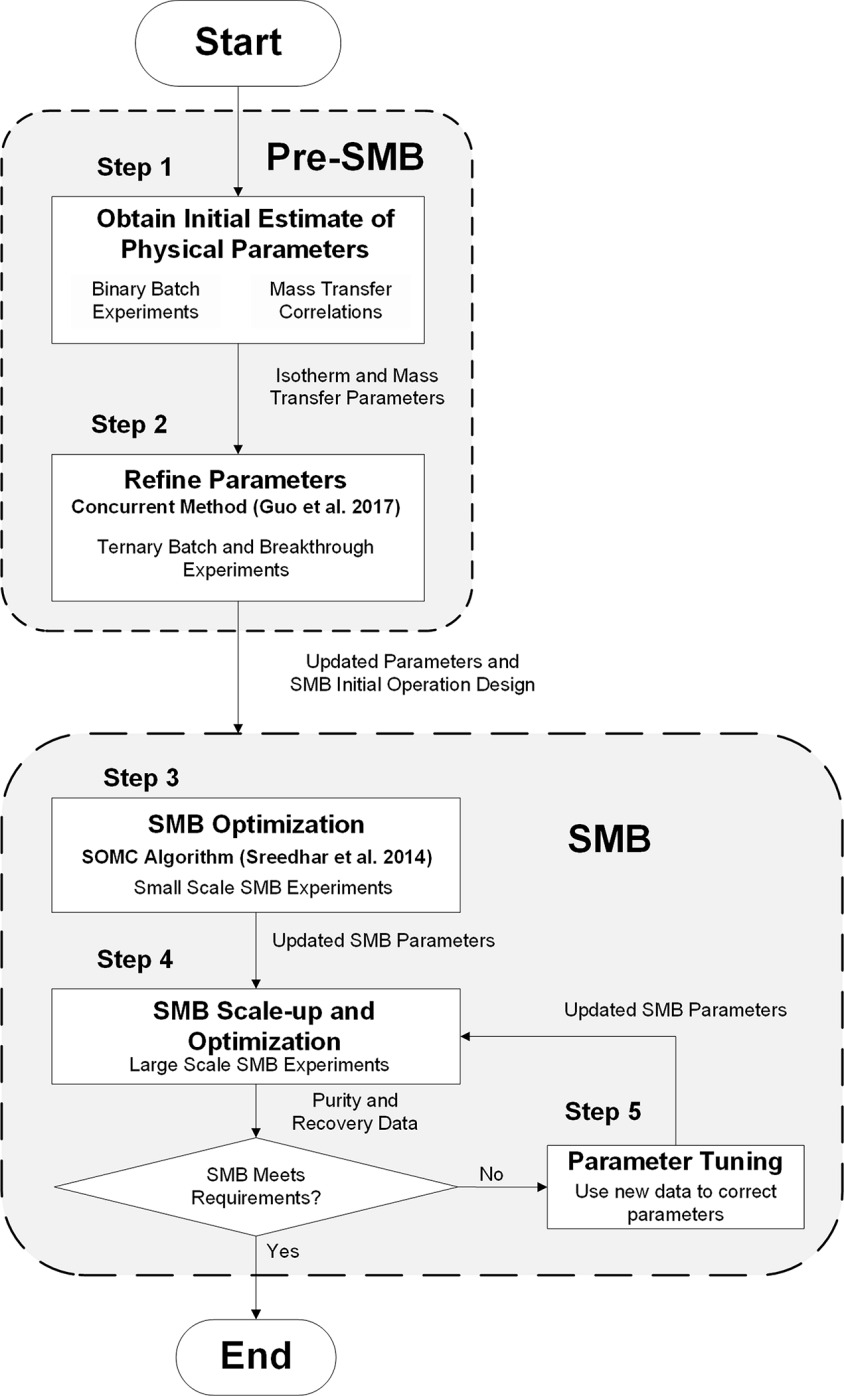
Framework for the model-guided SMB design and scale-up.

Figure 3 shows the stepwise approach used to design and scale-up the SMB system. This is an adapted version from a previous methodology used to design SMB experiments for hydrocarbon mixtures.^34^ As shown, this is a sequential approach, where the information obtained prior to any SMB run (Pre-SMB) is used to design the first SMB experiment and beyond (SMB). The algorithm assumes limited initial knowledge, where only information regarding the feed composition, adsorbent material, and desorbent is known. The result is an accurately parametrized large-scale SMB that recovers 2,3-BDO and meets all performance requirements (productivity, purity, and recovery). The Supporting Information (section S1) has a detailed nomenclature list of symbols and their associated quantities.

#### Pre-SMB Methods

Step 1: obtain an initial estimate of all physical parameters pertinent to the SMB. The isotherm parameters (*q*_m_, *K* and *H*) were obtained from fitting the mixed linear + Langmuir (MLL) model to data from batch adsorption experiments of binary mixtures (2,3-BDO/water and ethanol/water). The mass transfer data (*k*_app_ and Pe) were estimated from correlations found in the literature.^35−37^

Step 2: then, a set of ternary experiments (detailed conditions in Table S26) was designed based on the concurrent method proposed by Guo et al.^38^ After the experiments were conducted, the isotherm parameters were refitted using this new data. Breakthrough experiments were also used to calibrate the parameters.

#### SMB Methods

Step 3: the “Simultaneous Optimization and Model Correction” (SOMC) algorithm developed by Sreedhar and Kawajiri^39^ was used to guide the design of SMB experiments. This is an iterative approach, where the operating conditions (zone velocities and step time) of an experiment are selected based on the optimization of the SMB model. The experiment is conducted, and the parameters are tuned by fitting the model to the collected concentration data. Based on the updated parameters, a new set of operating conditions are obtained, and the process is iterated until the convergence criterion is met. At this point, only “small scale” (65 g of adsorbent) SMB experiments were conducted, with a binary 2,3-BDO (10 wt %) and water as the feed and pure ethanol as the desorbent. Details about the optimization and fitting framework are in Table S11 in the Supporting Information.

Step 4: the SMB is scaled to approximately seven times (∼520 g of adsorbent) the previous size. The operating conditions of this larger-scale system (Table S16) are obtained by optimizing the model based on the converged set of parameters from the previous step. The minimum performance requirements of this system are 0.20 kg_BDO_/day productivity at 70% desorbent-free purity and 95% recovery. The experiment is conducted, and if the results match the model, the algorithm is terminated. The feed to this SMB is the real broth with ∼10 wt % 2,3-BDO. Pure ethanol is still used as the desorbent.

Step 5: if the minimum performance requirements are not met, the model is tuned based on the new experimental data, and the updated parameters are used to repeat step 4.

#### Adsorption Breakthrough Measurements

The breakthrough experiments were carried out using stainless-steel columns packed with the adsorbent at 303 K. Ethanol was selected as the desorbent in this work due to its good miscibility with both 2,3-BDO and water and its low boiling point. Before the breakthrough measurements, the packed column was regenerated with 0.2 mL/min ethanol under 303 K for 500 min. In the breakthrough measurements, the feed solution (model broth or real pretreated broth) was introduced into the packed column using an HPLC pump (Shimadzu LC-20AD) at 0.2 mL/min. The outlet stream of the column was collected periodically into 2 mL HPLC vials in the fraction collector (Shimadzu FRC-10R). These samples were analyzed offline to obtain the points on the breakthrough curve at a specific time. The concentrations of sugars, alcohols, organic acids, and acetoin were analyzed by HPLC. The concentration of water was analyzed by GC. Due to the large molecule size and high hydrophilicity, maltose in the feed solution was assumed as the nonadsorbing component (tracer) in the breakthrough experiments. The Supporting Information (Section S2.3) describes our analysis confirming that maltose is a nonadsorbing penetrating tracer. The uptake of component i (*q*_i_, mg/g adsorbents) at a specific time *t* in the breakthrough measurements was calculated by
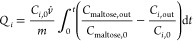
1where *m* (g) is the loading of the adsorbents in the column; *v̇* (mL/min) is the flow rate of the feed solution; *C*_*i*,0_ (g/L) is the concentration of component *i* in the feed stream and *C*_*i*,out_ (g/L) is the concentration in the outlet streams; the ratio of *C*_maltose,out_ (g/L) and *C*_maltose,0_ (g/L) is the normalized concentration of maltose in the outlet streams; *t* (min) is the duration of adsorption. Separation factors for pairs of components in the broth mixture were calculated as
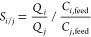
2where *Q*_*i*_ and *Q*_*j*_ are the adsorbed amounts of component *i* and *j* (mg/g adsorbents), whereas *C*_*i*,feed_ and *C*_*j*,feed_ are the concentrations of component (or species) *i* and *j* in the feed stream (g/L).

#### Cyclic Column Operation

Cyclic operation (back-to-back production runs) was performed on the GT-MFI column with three steps in each cycle. The first step is adsorption with 0.2 mL/min real pretreated broth as the feed stream, and the outlet stream was collected as BDO-free stream (raffinate). The adsorption was stopped at the breakthrough point of 2,3-BDO. The second step is the purge step to remove the liquid in the interstitial space between the adsorbent pellets. N_2_ was applied as the purge gas from gas cylinder at 50 mL/min controlled by flow meter for 0.5 h. The outlet stream can be recycled, as the composition is similar to the feed stream. However, the recycling of the interstitial stream is not investigated in this work. The third step is to desorb and regenerate the column with pure ethanol at a flow rate of 0.2 mL/min. In this step, the outlet stream collected is regarded as the extract product. The outlet stream during adsorption and desorption was collected periodically into the HPLC vials and analyzed offline. We defined the purity (wt %) in an ethanol and water free basis as
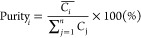
3where  is the average concentration (g/L) of component of interest (2,3-BDO in this work) and the sum at the denominator is the total average concentration of components other that water (i.e., sugars, organic acids, alcohols, and acetoin) in the stream. Recovery is another important indicator to evaluate the efficiency of the adsorption process. The outlet stream during the purge step could be recycled. The extract collected during desorption is the product stream, and the raffinate is where the loss of BDO occurred. Therefore, the recovery of component *i* in the production run can be calculated as
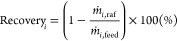
4where  and  are the mass productivity rate (kg·hr^–1^·ton^–1^ MFI adsorbents) of component *i* in raffinate and feed, respectively. The component *i* investigated in this work was 2,3-BDO. The productivity (g) of BDO during desorption can be calculated as
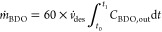
5where  (mL/min) is the flow rate of ethanol; *C*_BDO,out_ (g/L) is the concentration of BDO in the outlet stream; *t*_0_ (min) is the time when the extract product collection starts, and *t*_1_ (min) is the time when extract product collection ends.

#### Vacuum Distillation

Ethanol in the obtained BDO-rich extract streams from the desorption stage was recovered by vacuum distillation with a rotatory evaporator (Across International SE05). It was operated at 50 °C and 0.2 bar.

#### Batch Adsorption

To obtain estimates of the isotherm parameters, batch adsorption experiments were conducted for 2,3-BDO/water and ethanol/water binary mixtures at 296 K. Approximately 0.3 mg of MFI zeolite pellet with the appropriate amount of solution was added to a 20 mL glass vial. The solution volume to adsorbent mass ratios varied from 13 to 16 mL/g. The vials were shaken at 136 rpm on a digital platform shaker (New Brunswick Innova 2000) for 24 h at 296 K to ensure the adsorptions reached equilibrium. Then the supernatant solutions were filtered and transferred to a 1.5 mL glass vial (Supelco) through a 1 mL tuberculin syringe (BD) with a 0.2 μm syringe filter (Shimadzu) for concentration analysis. The following mass balance expressions are used to determine the adsorption uptake of each component.
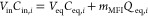
6where *C*_*i*_ is concentration of a component *i* in the solution in g/mL, *Q*_eq,*i*_ is the adsorption uptake in g/g_MFI_, *V* is the volume of the solution in mL, and *m*_MFI_ is the mass of the MFI adsorbent in g. The subscripts in and eq denote the initial and equilibrium states, respectively. In practice, *V*_eq_ and *Q*_eq,*i*_ cannot be measured directly from experiments, yielding *n* + 1 unknowns for *n* independent equations, where *n* is the number of components. Thus, an additional relation must be included to obtain a unique solution. As proposed by DeJaco et al., the following expression can be used
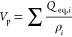
7

This is denoted as the pore filling adsorption model,^40^ where the adsorbed solution is assumed to behave like an ideal mixture and occupy a pore volume *V*_p_ (cm^3^/g_MFI_). In the case of the proposed MFI, it was assumed that *V*_p_ corresponds to the micropore volume of the adsorbent measured by physisorption (0.180 cm^3^/g_MFI_) and that ρ_*i*_ is the density of each component.

The collected batch adsorption data was used to obtain isotherm parameters that would then be incorporated to a dynamic adsorption model. The MLL isotherm was chosen for this system, where *q*_*m*,*i*_, *K*_*i*_, and *H*_*i*_ are the saturated capacity, Langmuir affinity constant, and Henry’s linear constant of each component *i* at equilibrium, respectively.
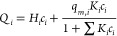
8

#### Liquid Sample Analysis

HPLC was used to quantitatively analyze the sugars (i.e., maltose, xylose, and arabinose), alcohols (i.e., glycerol, xylitol, and 2,3-BDO), organic acids (i.e., malic acid, lactic acid, and acetic acid), and acetoin in the model solution and real pretreated broth. A Shimadzu HPLC system was equipped with a Bio-Rad Aminex HPX87-H column (300 mm × 7.8 mm i.d.) at 65 °C. The mobile phase was 5 mM H_2_SO_4_ in DI water at a flow rate of 0.5 mL/min. The column was coupled to a refractive index detector. The sum concentration of arabinose and xylitol was calculated due to peak overlapping. The concentration of organic acids indicates the total concentration of their protonated forms and ionic pairs. Water was quantified by GC equipped with a Phenomenex ZB-1 column and a thermal conductivity detector.

#### SMB Modeling

SMB was modeled through the solution of a system of partial differential algebraic equations. The transport dispersive model proposed by Wu et al.^35^ was implemented to describe the SMB, and following the guidelines of Kawajiri and Biegler,^33^ a system of PDAEs was fully discretized in time and space and solved simultaneously with the appropriate cyclic-steady state (CSS) conditions. The result is a nonlinear programming (NLP) problem, which was built in Pyomo 6.6.2. and solved using IPOPT_sens 3.12.13.0. The following are the SMB equations:

Mass transfer in the solid (adsorbent) phase
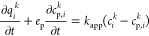
9

Mass transfer in the bulk liquid phase
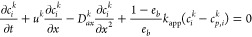
10

Adsorption equilibrium
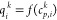
11where *q*_*i*_ is the concentration in the solid phase of the MFI adsorbent (g/cm^3^_MFI,bulk_) and is obtained by multiplying the adsorption uptake expression from eq 8 by the MFI adsorbent bulk density (*q*_*i*_ = *Q*_*i*_ × ρ_MFI,bulk_). Furthermore, *c*_p,*i*_ is concentration in the particle pores (g/cm^3^_solution_), *c*_*i*_ is the concentration of the bulk liquid (g/cm^3^_solution_), *e*_p_ is porosity of adsorbent pellets, *k*_app_ is apparent mass transfer coefficient (min^–1^), *u* is the superficial velocity (*v̇* = *u* × area) (cm/min), *D*_ax_ is the axial dispersion (cm^2^/min), and *f* is an adsorption isotherm expression that is used to obtain the uptake (instantaneous equilibrium is assumed). The subscript *i* denotes the components and the superscript *k* the SMB columns.

As a simplification, the dead volume of the system was represented by thin empty tubes positioned at the end of each adsorption bed column. These tubes are identical in size and are described by the following expression.
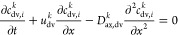
12

Finally, the boundary and cyclic state conditions were added to the model. These expressions can be found in the Supporting Information (eqs s17–s34).

#### SMB Operation

A SMB “mini-plant” unit (CSEP C190, Knauer) is used for experiments and production runs. As shown in Figure S5, the unit is equipped with four-piston pumps for controlling the inlet and outlet liquid flow streams of the system. A UV detector is connected to the outlet of the extract stream for online concentration monitoring. All eight adsorption columns (300 mm length and 20 mm inner diameter), each loaded with 65 g MFI adsorbent pellets (400–595 μm), are connected to the ports of the rotary valve. Figure S5 also shows the schematic diagram of the SMB where columns are connected in series, with two columns in each zone to form a 4-zone SMB configuration (2–2–2–2). The SMB operates in a countercurrent flow pattern. The column movement can be simulated by the rotary valve which switches the columns’ position relative to the inlet/outlet position per step time per column position. In this configuration, zone 2 and zone 3 act as the separation zones, where the strongly adsorbed component is selectively absorbed onto the adsorbent, while the weakly adsorbed components are carried through. Separation performance metrics for SMB are defined as follows
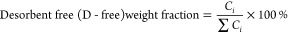
14
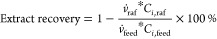
15
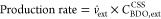
16

Here, we define extract stream purity as D-free weight fraction for component *i*, hence ∑*C*_*i*_ is concentration for all components excluding ethanol,  and  are the volumetric flow rate (mL/min) for raffinate stream and feed stream, *C*_BDO,ext_^CSS^ is the CSS average concentration (g/L) of 2,3-BDO in the extract stream.

## Results and Discussion

**Figure 4:**
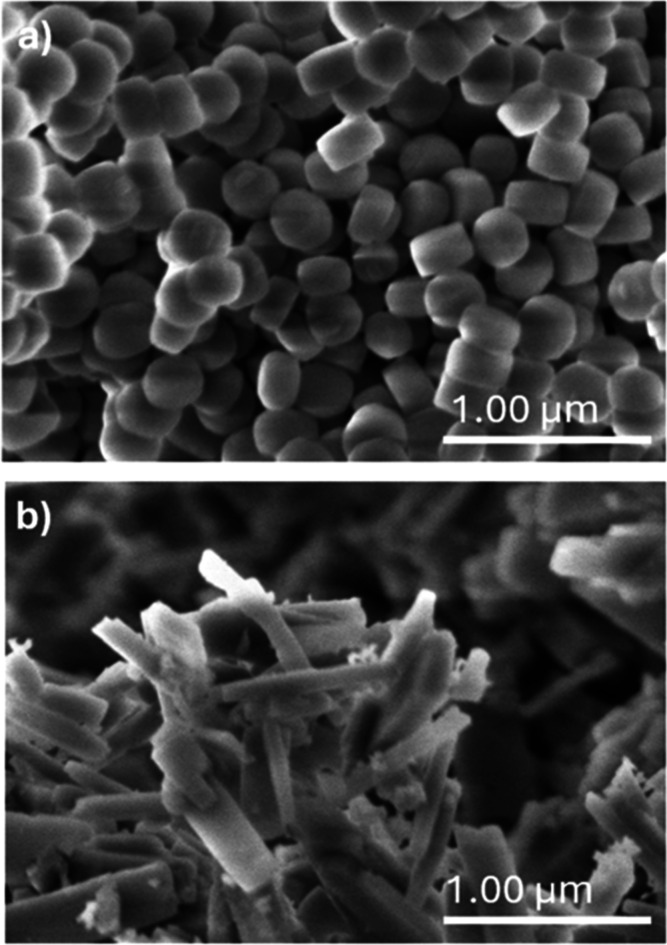
SEM images of (a) synthesized GT-MFI and (b) commercial cMFI crystals.

Figure 4a shows the morphology of the synthesized pure-silica MFI (GT-MFI) nanocrystals, which have a uniform pill-like shape and are ∼250 nm in diameter. The commercially available high-silica ZSM-5 (cMFI) is shown in Figure 4b and has a very different rod-like morphology, with lengths of 600–800 nm and thickness typically less than 100 nm. While Figure S1 (powder XRD) shows the presence of crystalline MFI-type zeolite in both materials, the physisorption isotherms are different (Figure S2). Specifically, Table S2 shows that the BET surface area and micropore volume of GT-MFI are considerably higher than those of cMFI.

**Figure 5:**
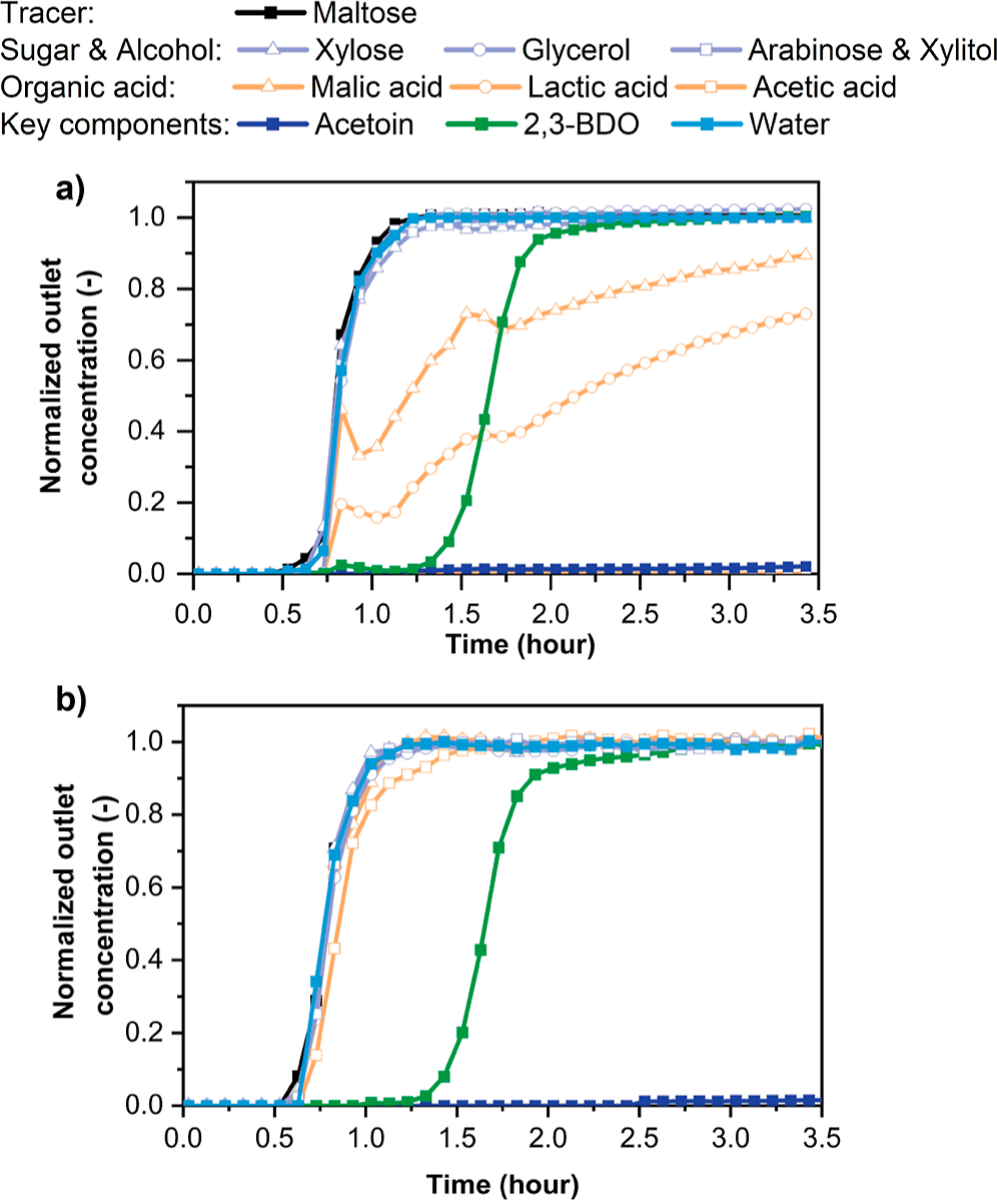
Multicomponent breakthrough experiments using (a) acidic model broth and (b) pH-neutral model broth. Both experiments were performed at 303 K using GT-MFI adsorbent and ethanol as desorbent. The outlet concentration of each component is normalized by its feed concentration.

Due to the presence of organic acids in the fermentation product broth, it is necessary to determine an appropriate pH to which the feed should be preadjusted before adsorptive separation. A model broth was prepared according to the composition shown in Table S3, which closely follows the composition of the main components in the fermentation broth. Due to the organic acids, the pH of this model feed was 2.4. A breakthrough measurement was performed with this feed at 303 K using a pelletized GT-MFI adsorbent column (ID 0.94 cm, length 20 cm), as shown in Figure 5a.

Maltose—a large disaccharide molecule, is considered as the tracer species that is assumed to be nonadsorbing in MFI. The sugars (xylose, arabinose), xylitol, glycerol, and water break through quickly along with maltose; i.e., they were not adsorbed due to their large molecular size and/or hydrophilicity. In contrast, 2,3-BDO and acetoin show strong adsorption in GT-MFI, with acetoin still not having broken through on the time scale of this experiment (due to its low concentration in the feed). This shows the excellent potential of MFI adsorbents for separating 2,3-BDO (and the closely related molecule acetoin) from the broth. However, acetic, lactic, and malic acids are also considerably adsorbed and have not broken through fully during the time scale of the experiment. With dissociation constants (p*K*_a_) in the 3.8–5.2 range for these acids, they exist substantially in protonated form at the feed pH of 2.4 and compete for adsorption sites with 2,3-BDO and acetoin. Therefore, the feed pH is adjusted to neutral with the addition of sodium hydroxide, allowing organic acids to be deprotonated into their ionic forms. This leads to a very sharp separation of 2,3-BDO and acetoin from all of the other components (Figure 5b).

**Figure 6:**
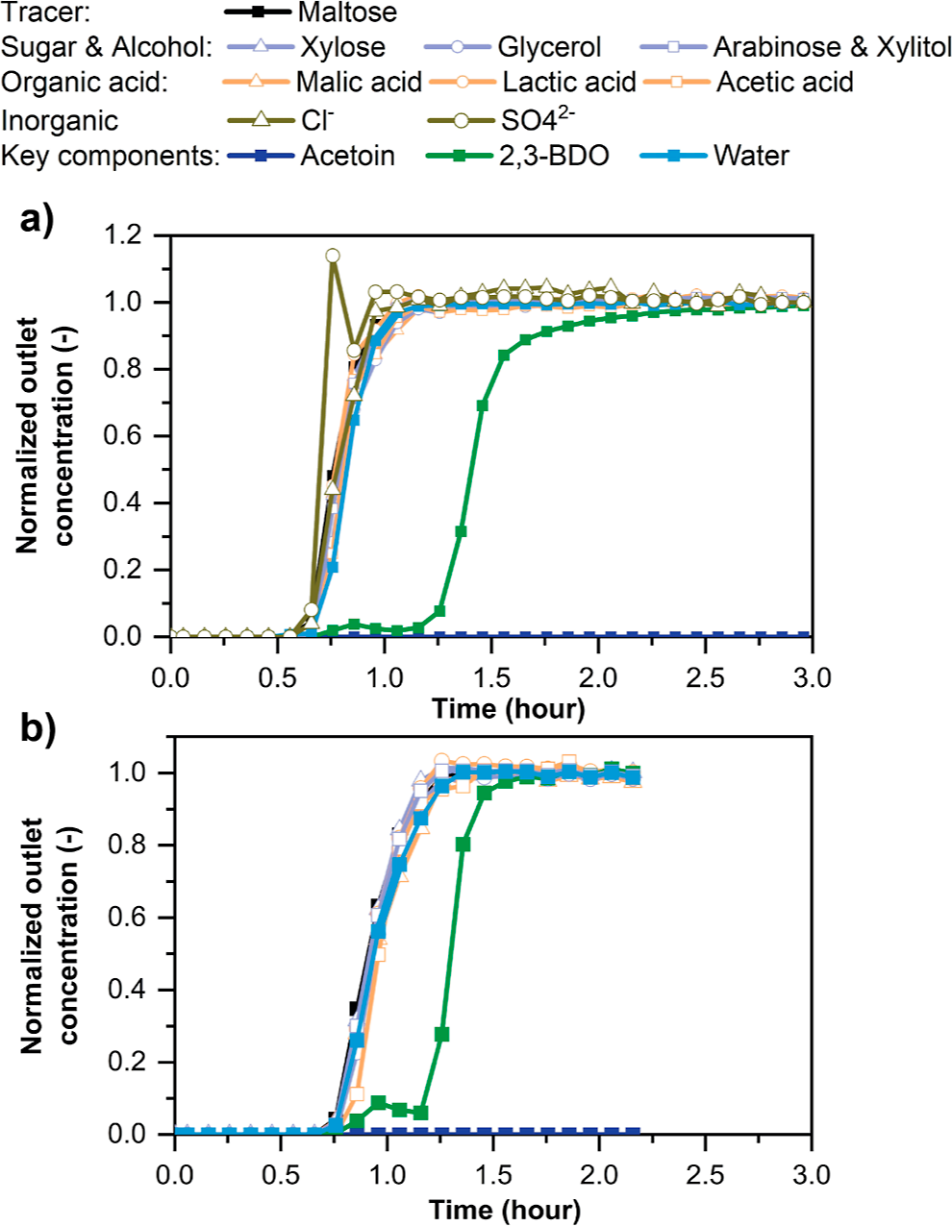
Multicomponent breakthrough experiments using on (a) GT-MFI and (b) cMFI columns at 303 K using real pretreated broth as feed and ethanol as desorbent.

The separation characteristics of the pelletized GT-MFI and cMFI adsorbents were then evaluated by breakthrough measurements on 20 cm-length packed columns at 303 K using the real pretreated fermentation broth (Figure 6 and Tables 1 and 2). Figure 6a shows a very sharp separation of 2,3-BDO and acetoin from all other components, including water, by the GT-MFI column. The “roll-up” like breakthrough behavior of the SO_4_^2–^ ion may be attributed to transient precipitation of inorganic salts due to low solubility in ethanol. Given the biomass-derived broth, cations such as Ca^2+^, Mg^2+^, Na^+^, and K^+^ would be expected to be present in small amounts. More detailed investigation of such potential phenomena is needed. A weak initial plateau is seen in the breakthrough curve of 2,3-BDO, which is likely caused by a small amount of mesoporosity (or, alternatively, external surface sites) existing in the pelleted GT-MFI adsorbent. This saturates faster than the microporosity due to the faster diffusion and uptake in these mesopores (or external surface sites).

**Table 1:** Equilibrium Uptakes of Each Component in GT-MFI and cMFI Columns, as Obtained From the Breakthrough Measurements (Figure 6) with Real Pretreated Fermentation Broth^a^

component	feed concentration (g/L)	uptake on GT-MFI (g/kg zeolite)	uptake on cMFI (g/kg zeolite)
maltose	8.7	0.0	0.0
xylose	2.4	<0.1	<0.1
arabinose & xylitol	7.4	<0.1	<0.1
glycerol	6.8	0.6	0.5
malic acid	3.0	<0.1	0.2
lactic acid	2.2	<0.1	<0.1
acetic acid	1.4	0.1	0.1
acetoin	0.3	1.0^a^	0.9^a^
BDO	100.2	92.7	68.9
water	892.1	72.1	60.2
Cl^–^	0.2	<0.1	
SO_4_^2-^	0.2	<0.1	

a The calculated uptake of acetoin is not the equilibrium adsorption uptake because it does not reach steady state during the time scale of the experiments.

**Table 2:** Separation Factors for Pairs of Components in GT-MFI and cMFI Columns Based on Figure 5^a^

pairs	separation factor GT-MFI	separation factor cMFI
Cl^–^/2,3-BDO	7.26 × 10^–4^	
SO_4_^2–^/2,3-BDO	7.26 × 10^–4^	
BDO/(sugars + alcohols)	37	29
BDO/organic acids	26	11
BDO/water	11	10
acetoin/water	38	37

a The calculated separation factor acetoin is not the equilibrium value since it does not break through during the time scale of the experiments.

The uptakes of each component are listed in Table 1 and the key separation factors are summarized in Table 2. The GT-MFI column exhibits a high 2,3-BDO uptake capacity (93 g/kg) and excellent separation factors (11–38) for 2,3-BDO and acetoin over all other component types. Although the microporosity of pure silica GT-MFI adsorbent is hydrophobic, there is still a significant amount of water adsorbed due to its high chemical potential (concentration is 892 g/L) in the feed, large external surface area of the nanoparticle-based adsorbent, and possible coadsorption of water with 2,3-BDO in the micropores. These can be attributed to the presence of external silanol groups, whose concentration increases with the external surface area as the primary particle size decreases. At the same time, faster mass transfer is achieved with smaller primary particle sizes. We synthesized GT-MFI with a primary particle size of <300 nm which provides good mass transfer, as indicated by the wider separation window between 2,3-BDO and the other components (Figure 6a) compared to the cMFI adsorbent in Figure 6b. This is an example of the tradeoff between adsorbent hydrophobicity and faster mass transfer characteristics. The commercial material cMFI is also able to separate 2,3-BDO and acetoin from the broth, but the 2,3-BDO uptake (about 69 g/kg zeolite) and separation factor are considerably lower. Furthermore, Figure 6b highlights another disadvantage of cMFI in that 2,3-BDO experiences higher mass transfer resistance and breaks through faster than in GT-MFI. The tailored GT-MFI adsorbent is hence the desirable candidate in terms of 2,3-BDO recovery and enrichment from fermentation broth.

**Figure 7:**
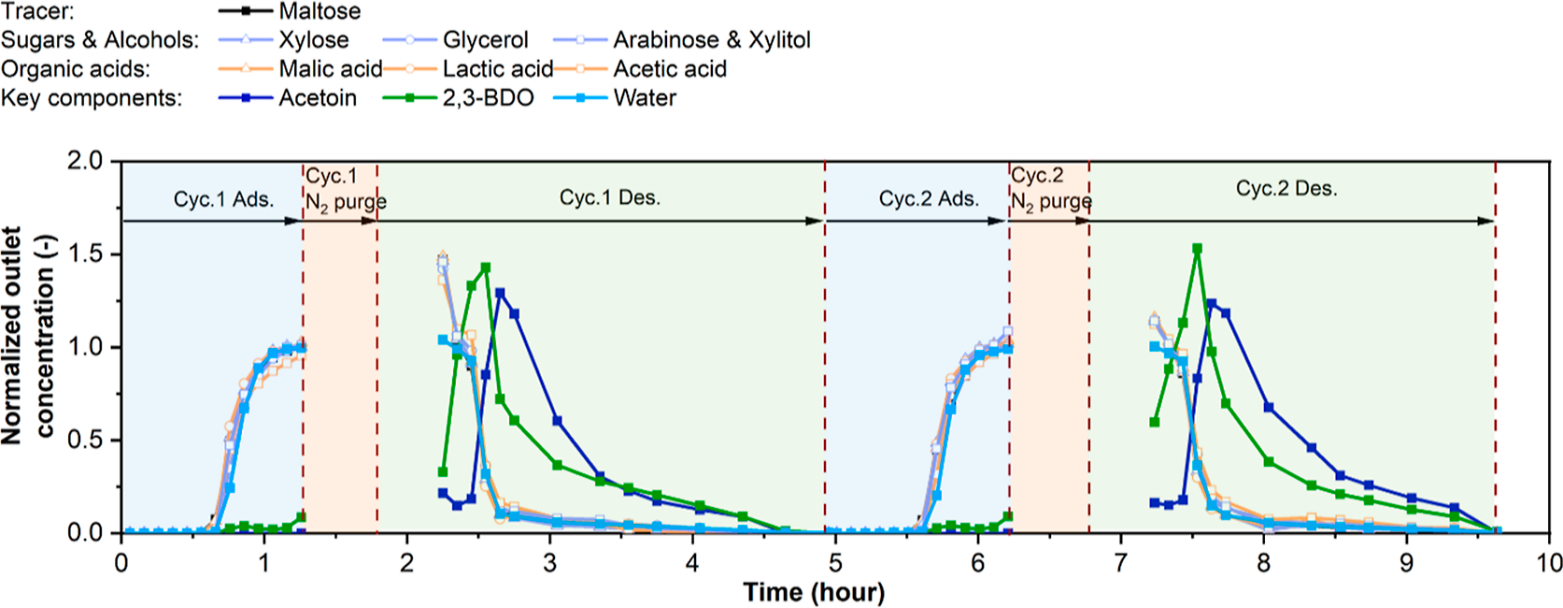
Cyclic operation of adsorptive 2,3-BDO recovery and enrichment from pretreated broth on a 20 cm GT-MFI column at 303 K. The adsorption, gas purge, and desorption steps are shown in blue, orange, and green regions, respectively.

Next, we performed cyclic adsorption experiments to evaluate the production of an enriched 2,3-BDO product stream from pretreated broth using the GT-MFI column for two back-to-back production cycles (Figure 7). Each cycle is divided into three steps (adsorption, purge, and desorption) and two cycles were performed to evaluate the robustness of the GT-MFI column.

Considering the adsorption step, the breakthrough behavior in Figure 6a shows that the breakthrough of most components is at about 0.7 h, while the concentration of 2,3-BDO increased sharply at about 1.3 h. To avoid significant loss of 2,3-BDO in the raffinate stream during adsorption, the pretreated broth feed was stopped after 1.3 h. The concentration profiles during the adsorption step (blue regions in Figure 7) exhibit the same trends as those in Figure 6a, and the uptake of each component during the adsorption in both cycles is calculated in Table S4. Because the equilibrium state is not required in the dynamic production runs, the adsorption uptakes are lower than those in the equilibrium breakthrough measurements. The purge step starts immediately after adsorption by switching the inlet stream to a N_2_ gas. By introducing a purge step before desorption, we can determine the quantity of the aqueous broth phase trapped in the interstitial porosity between the adsorbent pellets without desorbing the adsorbed phase in the zeolite micropores. The purge step lasted for 0.5 h (orange region in Figure 7) until no further aqueous liquid could be obtained at the column exit. Then the desorption step is started by switching the inlet stream to ethanol. In the first 0.4 h of desorption (green region in Figure 7), there is no outlet stream, as ethanol is filling the interstitial space. After this, the early stage of elution is contaminated with residual interstitial aqueous phase. As ethanol continues to pass through the column, 2,3-BDO and acetoin are increasingly displaced from the adsorbent, as indicated by the roll-up effect of the concentration profile of these two desired components. The desorption step is continued until 2,3-BDO and acetoin are completely displaced by ethanol. The same patterns are observed during both cycles, which indicates the proposed cyclic operation is robust and reproducible.

Ideally, the extract phase can contain more than 60 wt % BDO considering the uptake on GT-MFI during the adsorption step, based on the data shown in Table S4 (calculated from the cyclic production runs of Figure 7). The N_2_ purge step removes much of the aqueous liquid in the interstitial space between adsorbent pellets but not the aqueous liquid present in the macro-/mesopores within the adsorbent pellets. As shown in Figure 7, the contamination of the extract by the aqueous phase is seen in the early elution during desorption, wherein significant amounts of sugars, alcohols, acids, and especially water are present. Those concentration profiles are even higher than those of 2,3-BDO at the early stage. Therefore, it is possible to improve 2,3-BDO purity in the product stream by excluding early elution. Table S5 compares the separation performance and product quality by starting the extract product collection at different times during the desorption step. The BDO purity, recovery, and productivity are calculated as shown in eqs 3–5. By starting extract product collection later in the desorption step, the 2,3-BDO content and purity increase (due to lower contamination with the aqueous phase) whereas the 2,3-BDO productivity in the extract decreases due to the increased loss of 2,3-BDO in the contaminated initial elution stage. Therefore, there is a tradeoff between BDO purity and productivity. The total raffinate and extract collected from the two cycles were combined and their composition analyzed after removal of the ethanol desorbent by vacuum evaporation (which approximates a vacuum distillation column). The compositions of both streams are shown in Table S6. The concentration of 2,3-BDO increased from 99 g/L (feed) to 328 g/L (extract), which indicates that 2,3-BDO is enriched by about 3 times in the extract product stream. Furthermore, the gas purge step is impractical in large-scale applications. Finally, the total desorbent-to-feed ratio in each cycle is 3.34, which requires significant energy for ethanol desorbent recycling. As a result, a continuous countercurrent operation (which is approximated by an SMB system) is desirable to minimize the desorbent-to-feed ratio and increase the extract purity and 2,3-BDO recovery.

### Model Based Approach Implementation for SMB

**Figure 8:**
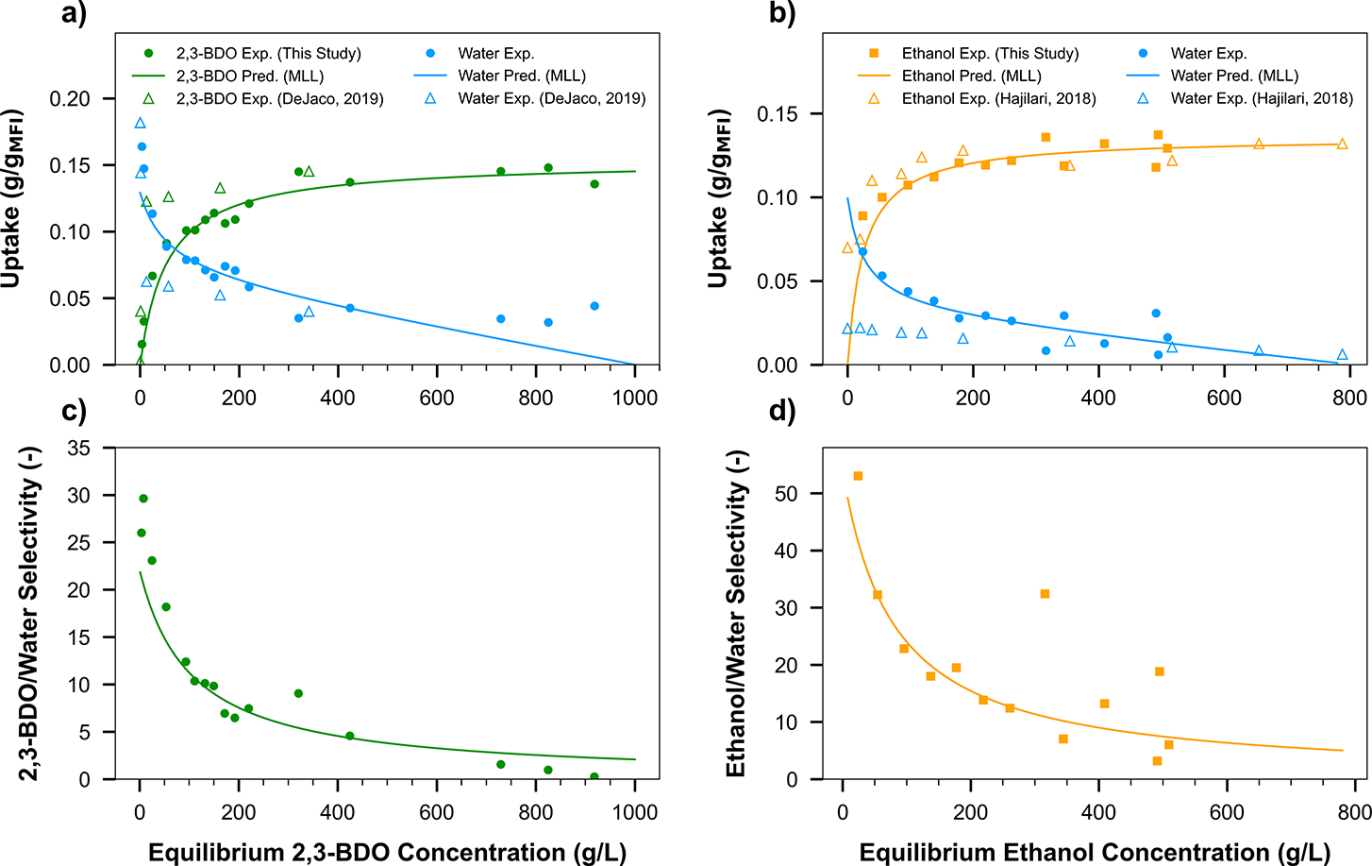
Equilibrium uptakes for (a) 2,3-BDO/water and (b) ethanol/water binary mixtures at 296 K and comparison to similar adsorbate/adsorbent systems found in the literature. 2,3-BDO/water selectivity (c) and ethanol/water selectivity (d). Symbols: experimental data, Solid curves: simulations (MLL model fits to uptake and selectivity). Literature data retrieved from DeJaco-Table S17 (323 K) and Hajilari-Figure 8 (303 K).

After obtaining insights from these cyclic adsorption runs, it was decided that SMB would offer a more efficient separation performance. The proposed model-guided approach was implemented to develop a predictive SMB model. Initially, batch adsorption experiments were conducted for 2,3-BDO/water and ethanol/water binary mixtures in GT-MFI (Figure 8) at 296 K. This was important for accurately predicting liquid and adsorbed phase compositions across four distinct zones and the column switching time. Figure 8 shows the obtained isotherms with the measured uptakes for 2,3-BDO and ethanol aligning with those reported for similar systems. The chosen MLL isotherm accurately predicts the uptake of these components.^40,41^Figure 8a,b shows the adsorption uptakes of 2,3-BDO/water and ethanol/water mixtures over a range of concentrations, and Figure 8c,d shows the corresponding 2,3-BDO/water and ethanol/water separation factors. The behavior of both mixtures is qualitatively similar. The adsorption uptakes and separation factors are very well fitted by the MLL model (eqn 8) as shown in Figure 8. The fitted MLL parameters (Table S7) revealed that ethanol exhibits a higher affinity constant (74.3 mL/g) to GT-MFI compared to 2,3-BDO (57.8 mL/g) as well as a higher separation factor from water. These characteristics, along with its low boiling point, make ethanol a very suitable desorbent. The affinity constants for water in both mixtures were very low (0.50 and 0.90 mL/g) due to the hydrophobic nature of the adsorbent, leading to high separation factors for the two alcohols over water. As expected with a fixed number of adsorption sites, the selectivities for 2,3-BDO and ethanol over water decline with increasing concentrations but remain >1 over most of the binary composition range. Next, the mass transfer parameters (Pe and *k*_app_) were estimated based on correlations previously used for SMB systems on aqueous mixtures.^35^Table S9 shows a summary of the estimated properties. The *k*_app_ values are well within the order magnitude reported by others for adsorption systems of miscible liquids^29,34,35,38,42^ and Pe was further validated by fitting of a tracer breakthrough curve. Further details of the calculation procedure can be found in the Supporting Information (eqs S9–S16).

These parameters were used to fit experimental breakthrough data for a 10 wt % 2,3-BDO feed at 303 K. Figure S3 shows the comparison between the predicted and experimental breakthrough. As observed, breakthrough times are accurately predicted, and there is good agreement from the model until the latter portion of the curve, where there are salient deviations. While some of this discrepancy can be attributed to the stability and precision of the chosen numerical solver, there are some aspects of the model (isotherm and mass transfer) that contribute to the deviation. First, the model predicts a roll-up effect that is not present in the experimental data, which is the result of using a competitive Langmuir isotherm to describe the uptake. Second, the experimental breakthrough curve for 2,3-BDO takes longer than expected to reach the saturation plateau compared to water. This trend is not captured by the model and can be attributed to the increased micropore transport resistance (i.e., slower diffusion) at higher 2,3-BDO loadings, thereby affecting the overall mass transfer of 2,3-BDO in the system. This is a behavior that has been observed for methanol in MFI and has been attributed to hindered diffusion as the particle becomes increasingly saturated.^43^ This was shown to decrease the methanol diffusivity by more than an order of magnitude. Despite the approximations of the present model, it is still able to predict the key features of the breakthrough curve. In addition, the concentration profiles observed in single adsorption columns are not expected to be like those in the SMB, and the deviations from the breakthrough curve are not significant enough to affect the applicability of the present model. Drawing from the conclusions of previous works,^34,44^ it was determined not to further refine the mass transfer parameters using breakthrough curves. The change in concentration (in both liquid and solid phases) observed in the breakthrough columns is not the same as in the SMB, and more accurate results can be obtained by fitting the SMB data directly.

**Figure 9:**
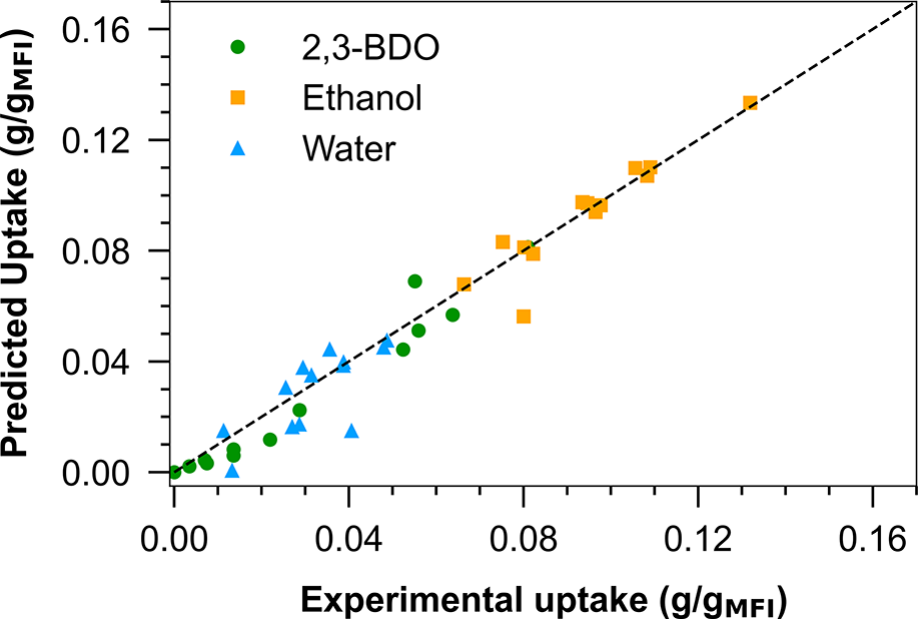
Parity plot comparing the experimental uptake of 2,3-BDO/ethanol/water ternary mixtures and the predicted uptake from fitting the MLL isotherm model (296 K).

Next, the concurrent approach was implemented. We developed an SMB profile that optimizes productivity based on the available parameters shown in Figure S6. This profile helped determine the composition of the ternary mixtures to be tested through batch experiments. After these experiments were conducted at 296 K, the MLL model was refitted to the data and a new set of parameters was obtained. Figure 9 shows the obtained parity plot for these experiments. As reported previously,^34,39,45^ even with a poor initial guess, the parameters can be calibrated more effectively by fitting the parameters to the SMB data directly. Therefore, only one iteration of the concurrent approach was implemented, and this set of parameters was used for designing the first SMB experiment.

#### SMB (Step 3)

Based upon the parameters listed in Tables S7 and S8, we conducted the validation and parameter tuning using a small-scale SMB equipped with eight 20 cm columns. The columns were individually tested for adsorption performance by conducting model solution breakthrough experiments under preparative chromatographic flow rate (2 mL/min). To ensure smooth operation of the preparative SMB, we increased the temperature from 303 to 323 K for the rest of this study to reduce liquid density and viscosity to prevent high pressure drop in the columns. Results showed consistent adsorption performance among the columns (Table S11), with a BDO uptake of 88 ± 3 mg/g of MFI and a BDO/H2O selectivity of 19 ± 5. Then, the eight columns were installed in our SMB unit for iterative process modeling and prediction. Four SMB experiments were conducted (detailed description can be found in Materials and Methods section), with model solution as feed and ethanol as desorbent. An adapted version of the SOMC algorithm was employed to design the SMB experiments at 323 K. Table S12 shows the converged parameters after four iterations. As seen, there is a deviation from these values, with respect to the pre-SMB estimates. Some of this discrepancy is expected, considering that temperature from the batch and SMB experiments is different and that the chosen mass transfer correlations can only provide an order of magnitude estimate. In addition, the large uncertainty in the uptake calculations of the batch experiments can also contribute to this deviation from the initial guess. Nevertheless, the converged parameters are still within a reasonable range.

**Figure 10:**
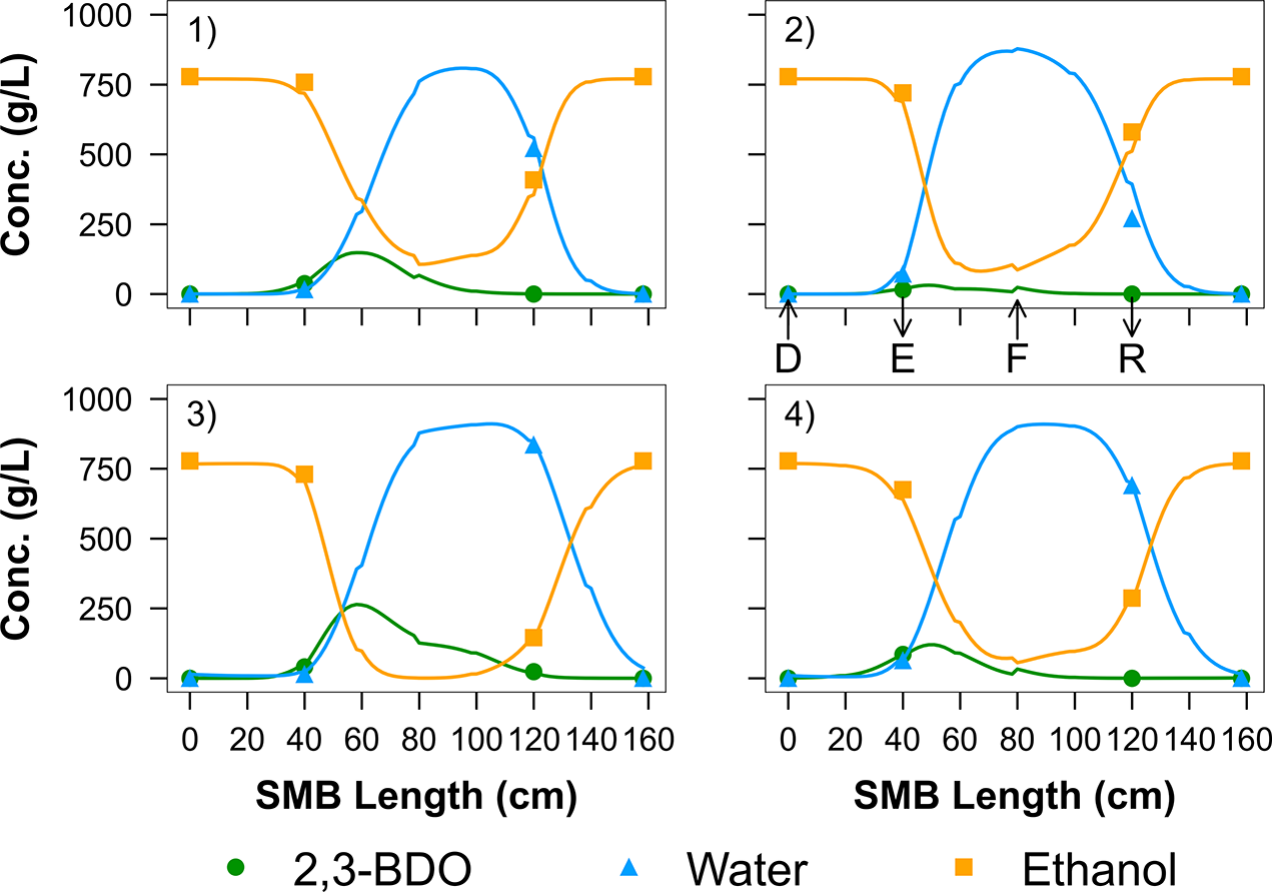
Predicted average internal concentration profiles at CSS for SMB experiments (1), (2), (3), and (4), using the converged set of parameters, *T* = 323 K. The feed is a binary 2,3-BDO (10 wt %)/water solution and ethanol is the desorbent. Operating conditions and concentration data of each experiment can be found in Tables S14 and S22–S25. Symbols: concentrations collected at the desorbent (D), extract (E), feed (F), and raffinate (R) locations, solid curves: predicted concentration profiles. Discontinuities in the predicted concentration profiles are due to the injection/removal of the input and output streams.

Figure 10 shows the average () SMB internal concentration profile predictions at CSS for the four runs, along with the experimental compositions at the desorbent (D), extract (E), feed (F), and raffinate (R) locations. The profile predictions closely match the experiments at these key locations. This also leads to accurate predictions of the main SMB performance parameters (productivity, purity, and recovery)^47^ as shown in the parity plot in Figure S4. Lastly, it is important to address the topic of parameter identifiability. As has been shown by others, ill-conditioning is unavoidable for highly nonlinear adsorption systems.^44,46^ This makes the parameters highly correlated and practically unidentifiable. Regularization is commonly used in these situations, and in this case, it helped us keep the parameters within a reasonable range. More small-scale experiments would also help improve the fitting procedure, but at this point, we decided to move onward to the scale-up of the system based on the converged set of parameters.

#### SMB Scale-Up (Steps 4 and 5)

Having established an initial SMB model and predictions for further scale-up, we then proceeded to modify the SMB system to validate the predictions. We scaled up the SMB unit by replacing the eight 20 cm columns with eight larger columns of 30 cm length and 2.1 cm ID, each filled with 65 g of adsorbent pellets, i.e., about 7.5 times higher adsorbent volume. The pellets used in these larger columns have a small amount (1.5–2 wt % of total pellet mass) of sodium silicate binder for increased mechanical strength to withstand the much higher flow rates. The binder was added to the prefabricated pellets. Table S20 shows a comparison of the separation characteristics before and after the addition of the binder. The 2,3-BDO uptake remains essentially identical, whereas the 2,3-BDO/water separation factor is somewhat lower after binder addition, likely due to the hydrophilic nature of the sodium silicate binder. However, the average separation factor remains well above 10. Therefore, we use the physical parameters shown in Tables S17 and S18 to solve the large-scale SMB model. As shown, for water, 2,3-BDO, and ethanol, we used the isotherm and mass transfer parameters obtained from the convergence of the small-scale SMB experiments. For acetoin, we used the same isotherm and mass transfer parameters of 2,3-BDO given their resemblance. For sugars, given their low concentration, we assumed the negligible values for the Langmuir terms (*q*_m_ and *K* ≈ 1 × 10^–5^) and a Henry’s constant of 0.10. We treated the inorganics as nonadsorbing components, and all isotherm parameters were set to very small (∼1 × 10^–5^) values. The remaining components (acids, xylitol, and glycerol) were treated as weakly adsorbed, and we used the same parameters of water, given their similar behavior in the breakthrough experiments. The apparent mass transfer coefficients of all the weak and nonadsorbing components were assumed to be the same as water.

**Figure 11:**
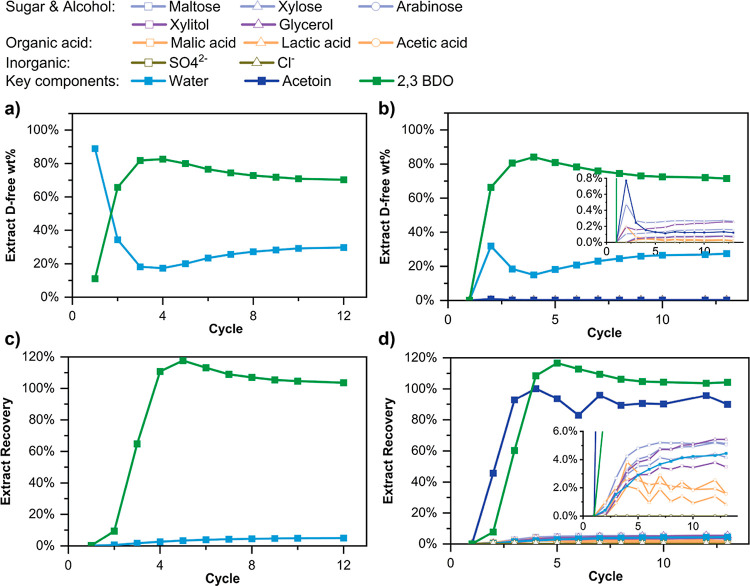
Evolution of the SMB performance metrics and approach to CSS in test runs: (a,c) run 1, desorbent-free extract stream composition and 2,3-BDO recovery in the extract; and (b,d) run 2, same quantities as run 1. The total run time was 690 min/12 cycles (run 1) and 744 min/13 cycles (run 2).

We performed two test SMB runs: the first uses a 10 wt % 2,3-BDO model feed whereas the second uses the model broth (Table S3). Both runs use the same set of operating conditions (Table S16) derived from the tuned SMB model, which predicts an extract with 77 wt % 2,3-BDO (desorbent-free basis), 98% recovery of 2,3-BDO in the extract, and a 2,3-BDO productivity of 0.345 kg/day in the extract stream. Figure 11 shows excellent results for both test runs. CSS is reached by cycle 9 wherein the extract composition becomes constant. The extract stream composition reached 71 wt % 2,3-BDO with a nearly 100% recovery of 2,3-BDO from the feed stream. After the CSS is reached, the productivity is 0.35 kg/day. Additionally, the other components in the model broth do not influence the enrichment and recovery of 2,3-BDO. Table S19 shows a comparison between the precited and experimental purity and recovery values of the components in the fermentation broth. These results clearly indicate that the model can accurately predict the SMB separation performance after increasing the scale.

#### SMB Production Run

**Figure 12:**
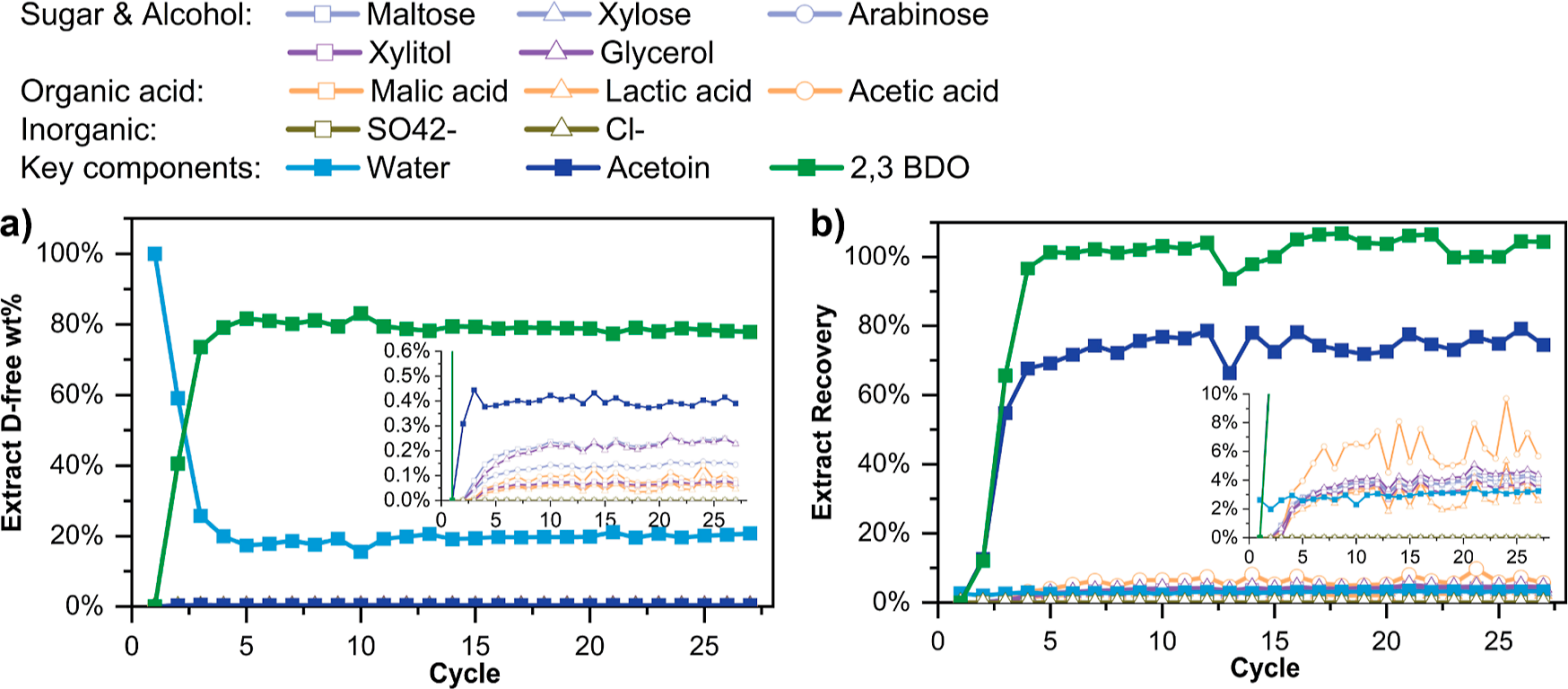
Transient evolution of the adsorption performance metrics for the SMB production run with the real fermentation broth: (a) composition of the desorbent-free extract stream (inset shows the minor components in more detail) and (b) recovery of individual components in the extract. The total run time is 1544 min/27 cycles.

We then performed a production run by feeding the pretreated fermentation process stream into the SMB. As seen in Figure 10, CSS was reached at cycle 4 with the extract stream reaching 80 wt % 2,3-BDO, the recovery of 2,3-BDO reaching nearly 100%, and productivity of 0.35 kg/day of highly concentrated 2,3-BDO (corresponding to a 2,3-BDO production rate of 28 kg/ton MFI/h). The cumulative extract product collected after the process reached CSS (i.e., cycle 4 onward) was analyzed after removing ethanol desorbent by vacuum evaporation. Table S21 shows the composition of the final product. The final 2,3-BDO concentration is 816 g/L, which is an 8-fold enrichment from the fermentation broth, with >98% purity. The concentrations of the other components are considerably decreased in the final product relative to those of the feed (Table S6), due to their very low recoveries in the extract (Figure 12b). To confirm the robustness of our system, we performed a second production run after regenerating the columns with the desorbent overnight at 2 mL/min. The same pretreated fermentation process stream was used as a feed, and the results are shown in Figure S8. The concentration profiles for 2,3-BDO, acetoin, and water in the extract stream are nearly identical, hence the separation performance of the adsorbent remains intact. This indicates that the engineered MFI adsorbent not only provides excellent separation performance but also mechanical and chemical robustness for realistic separations.

## Conclusions

The recovery and enrichment of 2,3-BDO from fermentation broth via adsorption on MFI zeolites have been investigated in detail. Preliminary breakthrough experiments using model broth with different pH values indicate that neutralization of the broth can deprotonate the organic acids (byproduct during fermentation), which facilitates their removal from 2,3-BDO through adsorption. Detailed column breakthrough experiments using both industrial MFI zeolite (aluminosilicate) and lab-scale synthesized pure silica MFI zeolite show that our synthesized MFI can provide higher BDO uptake, better BDO selectivities, and faster diffusion due to higher surface area and more uniform crystallization. The loading of 2,3-BDO from a pretreated fermentation broth (after filtration and neutralization) on the pure silica MFI zeolite can reach up to ∼93 g/kg of adsorbent. Detailed adsorption–desorption cycling of the MFI column shows robust and promising performance with the pretreated fermentation stream. Trade-offs between BDO purity and productivity by adjusting product stream collection during desorption is investigated. A concentrated aqueous 2,3-BDO product stream with 95% recovery, 93% purity, and 3-fold enrichment was achieved with a single column. On the other hand, a model-guided continuous adsorption (SMB) approach yielded a concentrated aqueous 2,3-BDO product stream with nearly 100% recovery, 80 wt % 2,3-BDO, and 8-fold enrichment. While cyclic adsorption offers simplicity in operation without requiring a robust mathematical model, SMB outperforms cyclic adsorption in terms of separation performance and emerges as a viable and scalable approach for the recovery and enrichment of 2,3-BDO.

## Data Availability

Data of the batch adsorption, breakthrough, and SMB (small and large scale) experiments are available in the Supporting Information.

## Supporting Information Available

Mathematical symbols and their associated quantities, detailed data on stream compositions and adsorbent characteristics; and structural and separation characteristics of adsorbents (PDF)
