# Supplementary material for: Separation of 2,3-Butanediol from Fermentation Broth via Cyclic and Simulated Moving Bed Adsorption Over Nano-MFI Zeolites
Source: ACS Sustain Chem Eng. 2024 Sep 12;12(38):14173–86. doi: 10.1021/acssuschemeng.4c04121 (PMC11423398; doi:10.1021/acssuschemeng.4c04121)
Supplement: Supplementary file 1 — sc4c04121_si_001.pdf [file sc4c04121_si_001.pdf]

Supporting Information

for

Separation of 2,3-butanediol from Fermentation Broth via Cyclic and SMB Adsorption over nano-MFI Zeolites

Jianpei Lao, Qiang Fu, Marco Avendano, Jason A. Bentley, Yadong Chiang, Matthew J. Realff, and Sankar Nair*

School of Chemical & Biomolecular Engineering, Georgia Institute of Technology
311 Ferst Drive NW, Atlanta, GA 30332-0100, USA

*** Corresponding author:** sankar.nair@chbe.gatech.edu

Number of pages: 40

Number of supporting tables: 27

Number of supporting figures: 8

Table of Contents

S1.Nomenclature……………………..……………………………………………………………S3

S2. Estimation of Physical Parameters…………………………………………………..S5

S2.1 Bed Porosity…………………………………………………………………..S5

S2.2 Particle Porosity………………………………………………………………S5

S2.3 Maltose as Tracer……………………………………………………………..S6

S2.4 Correlations for Mass Transfer Coefficient……………………………………...S7

S2.5 Transport Model Selection……………………………………………………S9

S2.6 Fitting of Isotherm…………………………………………………………...S10

S2.7 Fitting of the Breakthrough Curve……………………………………………..S11

S2.8 SMB Boundary and Cyclic Steady State Conditions……………………………..S12

S2.9 SOMC Optimization and Fitting……………………………………………….S13

S2.10 SMB Design Results………………………………………………………….S15

S3. Supplementary Tables……………………………………………………………………S17

S4. Supplementary Figures…………………………………………………………………...S34

S1. Nomenclature

Abbreviations

BDO	2,3-butanediol
CSS	Cyclic steady-state
IPOPT	Interior-point optimization
MFI	Mordenite framework inverted
MLL	Mixed linear + Langmuir
MOF	Metal-organic framework
NF	Nanofiltration
NLP	Non-linear programming
PDAE	Partial differential algebraic equations
PF	Pore filling
Pyomo	Python optimization modeling
SEM	Scanning electron microscope
SMB	Simulated moving bed
SOMC	Simultaneous optimization and model correction
TDM	Transport dispersive model
TEOS	Tetraethylorthosilicate
TPAOH	Tetrapropylammonium hydroxide
ZIF	Zeolitic imidazolate framework
ZSM-5	Zeolite Socony Mobil - 5

Mathematical quantities for SMB experiments and model

c_i^k	Concentration in the bulk liquid phase of component *i* in column *k*, g_{solute}/cm^3_{solute}
$c_{DV,i}^k$	Concentration in the bulk liquid phase of component *i* in the dead volume of column *k*, g_{solute}/cm^3_{solute}
$c_{p,i}^k$	Concentration in the pore space (meso+macropore) of component *i* in column *k*, g_{solute}/cm^3_{solute}
D_{ax}^k	Axial dispersion constant in column *k*, cm^2/min
H_i	Henry's linear constant of component *i*, $cm^3_{solute}/cm^3_{MFI,bulk}$
K_i	Langmuir affinity constant of component *i*, cm^3_{solute}/g_{solute}
k_{app}, i	Apparent mass transfer coefficient of component *i*, $1/min$
q_i^k	Concentration in the solid phase (adsorption uptake) of component *i* in column k, $g_{solute}/cm^3_{MFI,bulk}$
$q_{m,i}$	Langmuir saturation capacity of component *i*, $g_{solute}/cm^3_{MFI,bulk}$
u^k	Surface velocity of column *k*, cm/min
t	Time coordinate, min
t_{step}	SMB step time, min
u_{DV}^k	Surface velocity in the dead volume of column *k*, cm/min

$\dot{v}$	Volumetric flow rate, cm^3/min
x	Space coordinate
V_P	Micropore volume, $cm^3_{MFI,bulk}/g_{MFI}$

Mathematical quantities for batch adsorption experiments and models (static and breakthrough)

$C_{eq,i}$	Concentration in the bulk liquid phase of component *i* at equilibrium, g_{solute}/cm^3_{solute}
$C_{in,i}$	Concentration in the bulk liquid phase of component *i* at initial conditions, g_{solute}/cm^3_{solute}
m_{MFI}	Mass of adsorbent, g
$\dot{m}$	Mass productivity rate, $kg/hr/tonne_{MFI}$
$S_{i,j}$	Adsorbent separation factor (or selectivity) of component *i* over component j, (-)
Q_i	Concentration in the solid phase (adsorption uptake) of component i, g_{solute}/g_{MFI}
u	Surface velocity, cm/min
$\dot{v}$	Volumetric flow rate, cm^3/min
V_{in}	Volume of the solution at initial conditions, cm^3
V_{eq}	Volume of the solution at equilibrium, cm^3
V_P	Micropore volume, $cm^3_{MFI,bulk}/g_{MFI}$

Other symbols

ε_b	Bed (interstitial) Porosity
ε_p	Particle Porosity
ρ_i	Density of component i, g/cm^3
$\rho_{MFI,bulk}$	Bulk adsorbent (MFI) density, $g_{solute}/cm^3_{MFI,bulk}$

S2. Estimation of Physical Parameters

S2.1 Bed Porosity

The porosity of each column bed (ε_b) was estimated using the following expression. Where, m_{MFI} is the mass of MFI adsorbent packed in the bed, $\rho_{MFI,bulk}$ is the bulk density of the MFI, and Area and L are the area and length of the column. Thus, even though ε_b is unitless it can be expressed as (cm^3 of bed void volume)/(cm^3 of total bed volume) and represents the interstitial or interparticle volume inside the bed.

$$\varepsilon_b = 1 - \frac{m_{MFI}/\rho_{MFI,bulk}}{Area \times L} \tag{S1}$$

The bulk density of 0.945 g/cm^3 of the MFI was calculated by weighting MFI tablets (2.5 cm ID and 5mm thick) compressed using 1000 psi compression strength. This value was corroborated by the mercury intrusion porosimetry (MIP) test.

S2.2 Particle Porosity

The particle porosity of the adsorbent, ε_p (0.430), which is a key parameter in the dynamic model, was estimated based on the meso and macro pore volumes (V_{meso} = 0.18 cm^3/g_{MFI} and V_{macro} = 0.25 cm^3/g_{MFI}) estimated from MIP. It should be noted that ε_p is not the overall particle porosity, instead it is the fraction of the solid phase that is non-selective and is accessible to the tracer, which was used to successfully represent the breakthrough and SMB experimental data.

$$\varepsilon_p = (V_{meso} + V_{macro}) \times \rho_{MFI,bulk} \tag{S2}$$

Though unitless, ε_p can be expressed in $cm^3_{pore}/\ cm^3_{bulk,MFI}$, and represents the non-selective volume inside the particle, as opposed to the selective volume which is occupied by the micropores.

This value of particle porosity can be further tuned to improve the SMB model predictions. Furthermore, the obtained estimate of ε_p = 0.430 is an upper boundary. In practice, selective adsorption might occur in some of the small mesopores channels, and for modeling purposes it would be more appropriate to add this volume to the micropore space (represented by a saturation capacity q_m in Langmuir-like isotherms). Also, the pore volume accessible to the adsorbates might be smaller than the addition of meso- and macropore, further reducing the value of this ε_p estimate.

S2.3 Maltose as Tracer

To confirm that maltose was a suitable tracer, we evaluated its breakthrough data (**Figure S7**). The total porosity for a penetrating non-adsorbing tracer is given as $\varepsilon_T = t_{0,t}\frac{\dot{V}}{V_c}$, wherein ε_T is the total porosity, $\dot{V}$ is the volumetric flow rate (0.2 cc/min), V_c is the volume of the empty column (13.88 cc) and $t_{0,t}$ is the dead column time of the tracer. Then $t_{0,t}$ is estimated by subtracting, t_{plant}, the extra-column dead time, from t_R, the retention time of the tracer. The value t_R is the inflection point of the breakthrough curve. From the maltose breakthrough curve, the inflection point occurs at $t_R = 45.5$ min. The extra-column dead time is estimated using $t_{plant} = \mathrm{V}_{plant}/\dot{V}$, where V_{plant} is the extra-column dead volume has value of 0.5 cc, which was obtained with high accuracy by replacing the adsorption column with a zero-dead volume connection. This gives $t_{plant} = 2.5$ min and $t_{0,t} = 43.0$ min. All these parameters were obtained from direct measurements. Thus we obtain $\varepsilon_T = 0.62$. We then use $\varepsilon_T = \varepsilon_e + (1 - \varepsilon_e)\varepsilon_p$ to obtain the particle porosity, ε_p. ε_e is the bed or interstitial porosity. The bed porosity (ε_e = 0.343) was estimated based on the mass of adsorbent loaded to the bed (8.612 g) and the adsorbent bulk density ($\rho_{MFI,bulk}$= 0.945 g/cc), both of which were estimated with high accuracy in **Eq. S1**. Hence, we obtain $\varepsilon_p = 0.421$ and $V_p = \varepsilon_p/\rho_{MFI,bulk} = 0.444$ cc/g, which closely aligns with the addition of meso and macropore

volumes ($V_p = 0.43\ cc/g$) obtained via mercury intrusion porosimetry. This proves that maltose can enter the meso and macropore system (i.e. the non-selective volume) and is an indeed a penetrating tracer. Furthermore, the fact that ε_p only includes the mesopores and macropores also corroborates that maltose is non-adsorbing in the MFI adsorbent. For MFI the adsorption uptakes is based on the amount of a species that enters the micropore system. Thus, we can conclude that maltose is small enough to penetrate the meso/macropore volumes and large enough to be excluded from the micropores (the selective volume). This makes it a non-adsorbing penetrating tracer and a suitable candidate for measuring the selective adsorption uptake of other components. In addition, we further confirmed that maltose is a non-adsorbing and penetrating tracer by modeling the maltose breakthrough curve in **Figure S7** with the dynamic equations, specifically **Eq. 9** of the main manuscript with the assumption of a non-adsorbing component ($q_{maltose} = 0$). The mass balance in the liquid phase remains the same. As mentioned in the caption of **Figure S7**, the modeled breakthrough curve closely predicts the experimental data. Additionally, the fitted mass transfer coefficient and the Peclet number are within the values obtained from correlations.

S2.4 Correlations for Mass Transfer Coefficient

The following expressions were used to estimate the mass transfer coefficient:

$$k_{film,i} = \frac{3}{r_p} \times \left[\frac{1.09}{\varepsilon_b}\frac{D_{m,i}}{d_p}\left(\varepsilon_b \frac{v d_p}{D_{m,i}}\right)^{0.33}\right] \quad \text{(S3)}$$

$$k_{pore,i} = \frac{15\varepsilon_p D_{pore,i}}{r_p^2} \quad \text{(S4)}$$

$$D_{pore,i} = \frac{\varepsilon_p}{(2-\varepsilon_p)^2} D_{m,i} \quad \text{(S5)}$$

$$D_{m,i} = 7.74 \times 10^{-8} \frac{(\phi M)^{\frac{1}{2}}}{\mu V^{0.6}} \quad \text{(S6)}$$

$$k_{crystal,i} = \frac{15 D_{crystal,i}}{r_c^2} \quad \text{(S7)}$$

$$\frac{1}{k_{app,i}} = \frac{1}{k_{film,i}} + \frac{1}{k_{pore,i}} + \frac{1}{k_{crystal,i}} \quad \text{(S8)}$$

The first expression estimated the Wilson and Geankpolis correlation, which relates the Reynold's, Schmidt and Sherwood number, and solves for the film mass transfer coefficient, k_{film} (min^{-1}). Here, v (cm/min) is the interstitial velocity, and d_p, r_p, r_c , are particle diameter, particle radius, and the radius of the primary crystal, respectively, all in cm, and ε_b is the bed porosity (interparticle volume over total bed volume). $D_{pore,i}$ (cm^2/s), the pore or intraparticle diffusivity, is used to estimate the pore mass transfer coefficient, $k_{pore,i}$ (min^{-1}). $D_{m,i}$ (cm^2/s) is the bulk molecular diffusivity of component i in the mixture and ε_p is the particle porosity (macro- and mesopore volume over bulk MFI volume). $D_{m,i}$ is obtained from the Wilke-Chang expression, where ϕ is the solvent association factor, M is the solvent molecular weight (g/g-mol), μ is the solvent dynamic viscosity (cP), V_i is the solute molar volume (cm^3/mol) and T is the temperature in K. Even though Wilke-Chang applies only to dilute ideal mixtures, it was used because it can provided starting point and serve as an order of magnitude estimate of the mass transfer coefficient. This was further tuned through breakthrough and SMB experiments. Finally, $k_{crystal,i}$ (min^{-1}) is estimated based on $D_{crystal,i}$ (cm^2/s), the intra-crystalline diffusivity.[43] It is noteworthy that these expressions have been rearranged from Wu et al.[35] to forms that may be more familiar to the reader. **Table S9a – S9c** show the estimated ranges for the different mass transfer coefficients for 2-3,BDO, water, ethanol and maltose. The parameters for the Wilke-Chang expression were obtained from Taylor and Krishna,[36] while the estimates of intracrystalline diffusivity, D_c, were obtained for values reported by Karger et al.[43] for methanol/water, ethanol/water and n-butanol/water mixtures on MFI silicalites. Specific details of the estimation can be found on the github code. Note that parameters in **Table S9a – S9c** are only initial estimates, whereas the final set of fitting parameters is summarized in **Table S17**.

S2.5 Transport Model Selection

Selecting the appropriate model is crucial for ensuring the accuracy of the SMB predictions. The two most common transport models are the Linear Driving Force (LDF) model and Transport Dispersion (TD) model. **Equation S9-S10** shows that LDF simplifies the mass balances by using only one variable, q, to represent average concentration inside the particle (macro-, meso- and micropores).

$$\frac{\partial c}{\partial t} + \left(\frac{1-e_b}{e_b}\right)\frac{\partial q}{\partial t} + u\frac{\partial c}{\partial x} - D_{ax}\frac{\partial^2 c}{\partial x^2} = 0 \quad \text{(S9)}$$

$$\frac{\partial q}{\partial t} = k_{app}(q_{eq} - q) \quad \text{(S10)}$$

On the other hand, the TD model breaks down the concentration inside the particle into pore concentration c_p and adsorbed or solid phase concentration, q. The model assumes instantaneous adsorption, and k_{app} represents the mass transfer resistance from the bulk liquid to the particle pores. While LDF offers simplicity, TD provides a more nuanced understanding, capturing the behavior between fluid and pore phases, making it preferable for our system.

To determine their levels of accuracy, both models were used to fit maltose breakthrough data. Given that maltose size (~11 Å) is large compared to the medium pore size (~6 Å), maltose is assumed to be a non-adsorbing component and the solid phase q can be eliminated from the mass balance equations. **Figure S7** shows the comparison of the LDF and TD model for fitting the experimental tracer data. The model was solved using the following boundary and initial conditions, the dead volume is assumed to be a thin capillary tube located at the entrance of the bed adsorption column:

$$c_i(x, t = 0) = q_i(x, t = 0) = c_{p,i}(x, t = 0) = c_{dv,i}(x, t = 0) = 0 \quad \text{(S11)}$$

$$\frac{c_{dv,i}}{\partial x}(x = L, t) = \frac{c_i}{\partial x}(x = L, t) = 0 \quad \text{(S12)}$$

$$c_{dv,i}(x=L,t) = c_i(x=0,t) - \frac{D_{ax}}{u}\frac{c_i}{\partial x}(x=0,t) \tag{S13}$$

$$c_{dv,i}(x=0,t) = c_i - \frac{D_{ax}}{u_{dv}}\frac{c_{dv_i}}{\partial x}(x=0,t) \tag{S14}$$

As seen, the TD model can correctly fit the data, by only tunning $k_{app,maltose}$. On the other hand, LDF, whose only tunable parameter is Pe, gives unsatisfactory results. It is clear then, and expected, that maltose does transfer into the particles macropore space, and treating as a non-adsorbing component in LDF won't lead to an appropriate description of the system. To account for this transfer into particle without adsorption a linear isotherm could be assumed for maltose, and other non-adsorbing components, a reasonable assumption given that both models already used lumped parameters. However, the challenge lies on MLL isotherm already having a Henry's constant that is used to account for multilayer adsorption in the mesopores and lumping the two would not let us discern between the different scales (macro, meso and micro) within the particle. In addition, this would also lead to higher correlation between parameters, which would confound the fitting process in the more complex case of SMB. For all these reasons the TD was chosen for all future adsorption modeling.

S2.6 Fitting of Isotherm

As demonstrated by previous works from others,[28,35,47] the MLL isotherm can be used to accurately represent the adsorption of polar mixtures in SMB systems. In particular, Wu et al studied an aqueous dilute fermentation broth containing citric acid and reported agreement between experimental and predicted SMB purity using an MLL isotherm. Thus, this was determined to be a suitable model to describe the competitive adsorption inside the micropores of the MFI (Langmuir term) as well as the non-selective pore filling mechanisms inside the meso- and

macropores (linear term). An initial estimate of the MLL parameters was obtained by optimizing the following sum of squared errors (SSE) loss function:

$$\min_{\theta} \sum_n \sum_i w_n \left[q_{eq,i,n}^{exp} - q_{eq,i,n}^{pred}(c_{eq,i,n};\theta)\right]^2 \qquad \text{(S15)}$$

The parameters, θ, are tuned to minimize the SSE expression, the superscripts exp and pred indicate experimental and predicted values, respectively, and the subscript n, the number of the experiment. Each SSE term was weighted by w_n. This problem was solved using the Levenberg-Marquardt algorithm in the curve_fit module for SciPy in Python. Further details can be found in the github code.

S2.7 Fitting of the Breakthrough Curve

Next, the breakthrough curve data for 2-3,BDO and water (the main components in the feed) was fitted. The TDM model was applied to represent the column and dead volume. It is assumed that the area of the dead volume is 1/10th of the bed column area. The model was solved using the already mentioned equations and boundary and initial conditions and the following expression was optimized.

$$\min_{\theta} \sum_t \sum_i w_t \left[C_i(x = L, t) - C_{i,exp}(t)\right]^2 + \sum_m \lambda_m \left(\theta - \theta_m^{ref}\right)^2 \qquad \text{(S16)}$$

The first term is the sum of squared errors (SSE), where a vector of parameters θ is tuned to reduce the error between the predicted concentration at the end of the bed $C_i(x = L, t)$ and the experimental measured samples, $C_{i,exp}(t)$, at each the various sampling times t. We chose $k_{app,water}$, $k_{app,BDO}$ and D_{ax} as the set of tunable parameters. Furthermore, given the wide range in the concentration values, each term in the SSE is weighted by w_t. For samples at time s greater than or equal to breakthrough (samples with $C_i \geq 0.05\ C_i^{Feed}$), w_t is inversely proportional to the

measured outlet concentration value. For those below breakthrough time, w_t is the same as w_t at breakthrough. We introduced a vector of regularization weights, λ_m, which prevents the fitted parameters from largely deviating from θ_m^{ref} and becoming physically unreliable. As reference values, for D_{ax} we used the value obtained from the maltose curve and for $k_{app,i}$ the estimates from the correlations. More details about the SSE and regularization weights can be found in the code.

S2.8 SMB Boundary and Cyclic Steady State Conditions

Boundary Conditions

$$c_{0,i}^k = c_i^k(x = 0, t) - \frac{D_{ax}^k}{u^k}\frac{\partial c_i^k}{\partial x}|_{x=0} \quad \text{(S17)}$$

$$c_{dv,i}^k(x = 0, t) = c_i^k(x = L, t) \quad \text{(S18)}$$

Node Balances

Assume continuity $A \cdot u = A_{dv} \cdot u_{dv}$ between the thin tube (dead volume) and its corresponding adsorption bed column.

For columns 1 (first column in Zone I) and column 5 (first column in Zone III):

$$u^1 c_{0,i}^1 = u^8 c_{dv,i}^8(x = L_{dv}, t) + u^D c_i^D \quad \text{(S19)}$$

$$u^5 c_{0,i}^5 = u^4 c_{dv,i}^4(x = L_{dv}, t) + u^F c_i^F \quad \text{(S20)}$$

For all other columns:

$$c_{0,i}^k = c_{dv,i}^{k-1}(x = L_{dv}, t) \quad \text{(S21)}$$

Flow Balances

For columns connected to inlet and outlet ports

$$u^1 = u^8 + u^D \quad \text{(S22)}$$

$$u^3 = u^2 - u^E \quad \text{(S23)}$$

$$u^5 = u^4 + u^F \tag{S24}$$

$$u^7 = u^6 - u^R \tag{S25}$$

For all other columns

$$u^k = u^{k-1} \tag{S26}$$

Cyclic Steady-State Conditions

For recycle columns (columns 8 and 1)

$$c_i^8(x, t = 0) = c_i^1(x, t = t_{step}) \tag{S27}$$

$$c_{dv,i}^8(x, t = 0) = c_{dv,i}^1(x, t = t_{step}) \tag{S28}$$

$$c_{p,i}^8(x, t = 0) = c_{p,i}^1(x, t = t_{step}) \tag{S29}$$

$$q_i^8(x, t = 0) = q_i^1(x, t = t_{step}) \tag{S30}$$

For all other columns

$$c_i^k(x, t = 0) = c_i^{k+1}(x, t = t_{step}) \tag{S31}$$

$$c_{dv,i}^k(x, t = 0) = c_{dv,i}^{k+1}(x, t = t_{step}) \tag{S32}$$

$$c_{p,i}^k(x, t = 0) = c_{p,i}^{k+1}(x, t = t_{step}) \tag{S33}$$

$$q_i^k(x, t = 0) = q_i^{k+1}(x, t = t_{step}) \tag{S34}$$

S2.9 SOMC Optimization and Fitting

The SOMC requires a framework for both the SMB productivity optimization and sum of squares minimization routines. The following is the optimization framework, where PR represents the productivity (defined in the main body text), which is estimated by solving the system of PDAEs denoted by $\mathbf{f}(\vec{\mathbf{w}}; \boldsymbol{\theta})$. Furthermore, $\vec{\mathrm{w}}$ is the vector of input control variables and θ is the vector of

parameters. The solution of the system is also subject to minimum recovery and purity requirements:

$$\max_{\vec{w}} PR \tag{S34}$$

$$s.t.\, \boldsymbol{f}(\vec{\boldsymbol{w}}; \boldsymbol{\theta}) = 0 \tag{S35}$$

$$Recovery \geq Recovery_{min} \tag{S37}$$

$$Purity \geq Purity_{min} \tag{S38}$$

$$\vec{\boldsymbol{w}} = \left[u^{zone\ I}, u^{zone\ II}, u^{zone\ III}, u^{zone\ IV}, t_{step}\right]^T \tag{S38}$$

$$\boldsymbol{\theta} = \left[H_{BDO}, H_{water}, H_{EtOH}, q_{m,BDO}, q_{m,water}, q_{m,EtOH}, K_{BDO}, K_{water}, K_{EtOH}, k_{app,BDO}, k_{app,water}, k_{app,EtOH}, \varepsilon_p, DV\right]^T$$

(S39)

The sum of squares minimization framework was set-up similarly, by setting the followings expression as the objective function and constraints.

$$\min_{\theta} \sum_n \sum_i w_n \left[\left(\frac{\bar{C}^{model}_{ext,i,n} - \bar{C}^{exp}_{ext,i,n}}{\bar{C}^{exp}_{ext,i,n}}\right)^2 + \left(\frac{\bar{C}^{model}_{raff,BDO,n} - \bar{C}^{exp}_{raff,BDO,n}}{\bar{C}^{exp}_{raff,BDO,n}}\right)^2\right] + \sum_m \lambda_m \left(\theta - \theta^{ref}_m\right)^2 \tag{S40}$$

$$s.t.\, \boldsymbol{f}(\vec{\boldsymbol{w}}; \boldsymbol{\theta}) = 0 \tag{S41}$$

$$\boldsymbol{\theta_{lb}} \leq \boldsymbol{\theta} \leq \boldsymbol{\theta_{ub}} \tag{S42}$$

$$\bar{C}^{model}_{ext,i,n} = \int_0^{t_{step}} c^{k=1}_{i,n}(x = L, t)dt \tag{S43}$$

$$\bar{C}^{model}_{raff,i,n} = \int_0^{t_{step}} c^{k=3}_{i,n}(x = L, t)dt \tag{S44}$$

Where $\bar{C}^{exp}_{ext,i,n}$ and $\bar{C}^{exp}_{raff,i,n}$ are the experimentally measured average concentrations in the extract and raffinate, respectively, for a full SMB cycle. As shown above, these concentrations values were approximated in the model by the integrals solved using the trapezoidal rule. The subscripts

i and n indices denote the component and experiment number, respectively, and the superscript k the column number. In the case of the extract concentration only BDO and water were included in the set of measured variables, and for the raffinate, only that of BDO was included. This subset of measurement variables was chosen because it contains the only concentrations that are relevant to the predefined SMB performance metrics (productivity, purity and recovery). Similar to the breakthrough fitting, we included a regularization terms in the loss-function, where θ^{ref} represents the vector of fitted parameters from the previous iteration. This helps scale parameters with different magnitudes, and also prevent them from converging to unrealistic values. The optimization is quite sensitive to regularization term, so the regularization factors λ_m were ranged from 0.1 to 1.0. In each iteration, the problem was solved using multiple regularization factors, which in some instances led to different fitted parameters. The final set of parameters in each step was determined based on our judgement. Finally, θ_{lb} and θ_{ub} are vectors containing lower and upper boundaries of the parameters.

S2.10 SMB Design Results

Table S17 shows the evolution of the SMB parameters, starting with the initial guess (k=0) and the updated after each of the four experiments (k=1,2,3,4). After the first experiment (k=1), the model was able to be fit the data without major change to the initial guess, except for the Henry's constants, H. However, the obtained H of ethanol was very unrealistic and after the second experiment (k=2) the dead volume, DV, of each bed column was included into in the model. This lead to more realistic values for H but a drastic change in the K for BDO. This value is still within a reasonable order of magnitude, so the model was maintained. After the third experiment (k=3), a recovery of BDO of 75% was observed, which was the first time the recovery was incorrectly

predicted. Thus, the regularization terms were relaxed and some of the parameters changed more significantly. Nevertheless, these values are still within a reasonable order of magnitude and gave satisfactory fits. Lastly, for k=4 the fitting only led to minor changes in parameters and the algorithm was terminated. The solution for each of the iterations is provided in the github code.

The root-mean squared error (RMSE) was used to assess model accuracy using the following and was computed with the following expression. Where y_i denotes the measured variable from experiment i and n is the number of experiments. Superscripts exp. and model represent the experimental data and modeling predictions, respectively.

$$RMSE = \sqrt{\frac{\Sigma\left(y_i^{exp.} - y_i^{model}\right)^2}{n}} \tag{S45}$$

S3. Supplementary Tables

Table S1. The hydrophobicity properties and molecular size of the components in the fermentation broth and potential desorbents. The hydrophobicity is indicated by the n-octanol-water partition coefficient and the molecular size is compared by kinetic diameter (KD). These components are divided into different class, e.g., tracer, sugars, alcohols, organic acids, key components, and (potential) desorbents.

**Components**	**Formula**	**Species**	**log K_{OW}**	**KD (Å)**
Maltose	$C_{12}H_{22}O_{11}$	Tracer	-5.12	11.1
Xylose	$C_5H_{10}O_5$	Sugar	-3.02	7.9
Arabinose	$C_5H_{10}O_5$	Sugar	-1.98	8.1
Xylitol	$C_5H_{12}O_5$	Alcohol	-2.56	6.7
Glycerol	$C_3H_8O_3$	Alcohol	-1.76	6.3
Malic acid	$C_4H_6O_5$	Organic acid	-1.26	8.6
Lactic acid	$C_3H_6O_3$	Organic acid	-0.72	6.1
Acetic acid	$C_2H_4O_2$	Organic acid	-0.17	5.5
Acetoin	$C_4H_8O_2$	Key component	-0.36	5.5
2,3-BDO	$C_4H_{10}O_2$	Key component	-0.92	6.8
Water	H_2O	Key component	-1.38	2.9
Ethanol	C_2H_6O	Desorbent	-0.31	4.7
Methanol	CH_4O	Potential desorbent	-0.77	4.4
1-propanol	C_3H_8O	Potential desorbent	0.25	5.6
Isopropanol	C_3H_8O	Potential desorbent	0.05	5.6

Table S2. Textural properties of synthesized pure-silica MFI (GT-MFI) and commercial high-silica MFI (cMFI) pellets obtained from N_2 physisorption isotherms.

Characteristic	GT-MFI	cMFI
BET surface area (m^2/g)	467	364
Micropore Volume (cm^3/g)	0.18	0.14
Mesopore Volume (cm^3/g)	0.18	0.04
Median pore width (Å)	5.66	5.44

Table S3. Composition of model broth.

Component	Feed Concentration (g/L)
Maltose	12.9
Xylose	2.3
Arabinose	2.7
Xylitol	2.5
Glycerol	4.6
Malic acid	2.7
Lactic acid	2.7
Acetic acid	1.5
Acetoin	1.5
2,3-BDO	61.3
Water	932.0
Cl^-	0.3
SO_4^{2-}	0.2

Table S4. Uptakes of each component in the pretreated broth during the cyclic runs estimated using the breakthrough curves (**Figure 6**) measured with the GT-MFI column.

Component	Feed Concentration (g/L)	Uptake in Cycle 1 (g/kg zeolite)	Uptake in Cycle 2 (g/kg zeolite)
Maltose	8.1	0.0	0.0
Xylose	2.3	<0.1	<0.1
Arabinose & Xylitol	7.3	<0.1	<0.1
Glycerol	6.9	0.3	0.2
Malic acid	2.8	<0.1	<0.1
Lactic acid	2.2	<0.1	<0.1
Acetic acid	1.4	0.1	<0.1
Acetoin	0.3	0.2	0.2
2,3-BDO	98.9	65.3	61.6
Water	893.4	34.1	40.0

Table S5. Tradeoff analysis on the BDO purity and productivity from the cyclic back-to-back production runs, using pretreated broth with the GT-MFI column.

Cycle 1					
**Start time of extract collection after desorption step begins (h)**	0.40	0.50	0.60	0.70	0.80
**Extract BDO purity (ethanol & water free)**	86.6	88.3	91.1	93.3	94.9
**BDO productivity (g)**	1.13	1.11	1.04	0.90	0.61
**BDO (wt%)**	19.4	21.3	25.8	31.9	38.0
**Water (wt%)**	77.6	75.9	71.7	65.8	60.0
**Cycle 2**					
**Start time of extract collection after desorption step begins (h)**	0.40	0.50	0.60	0.70	0.80
**Extract BDO purity (ethanol & water free)**	87.7	88.8	91.0	93.2	94.5
**BDO productivity (g)**	1.17	1.13	1.05	0.93	0.77
**BDO (wt%)**	20.2	21.8	26.0	32.5	38.8
**Water (wt%)**	77.0	75.5	71.4	65.2	59.0

Table S6. Composition of the feed broth, ethanol-free 2,3-BDO extract product, and ethanol-free raffinate, during the cyclic back-to-back production runs.

Components	Concentration in the feed (g/L)	Concentration in the ethanol-free extract (g/L)	Concentration in the ethanol-free raffinate (g/L)
Maltose	8.1	5.3	9.1
Xylose	2.3	1.5	2.6
Arabinose & Xylitol	7.3	5.6	8.2
Glycerol	6.9	6.0	7.4
Malic acid	2.8	1.9	3.2
Lactic acid	2.2	1.6	2.6
Acetic acid	1.4	1.4	1.5
Acetoin	0.3	1.3	<0.1
2,3-BDO	98.9	328.5	13.1
Water	893.4	647.0	952.3

Table S7. Fitted parameters of the MLL isotherm for binary mixtures at 23 ℃.

Isotherm Parameter	2,3-BDO/Water		Ethanol/Water	
	2,3-BDO	Water	Ethanol	Water
q_m ($g_{adsorbate}/cc_{MFI}$)	0.140	0.118*	0.126	0.118*
Affinity Constant K ($cc_{solution}/g_{adsorbate}$)	38.4	1.00*	79.6	1.00*
Henry's Constant H ($cc_{solution}/cc_{MFI}$)	$6.57 x 10^{-4}$	$6.28 x 10^{-2}$	0.0	$2.97 x 10^{-2}$

* Fixed parameter

Table S8. N_2 physisorption and mercury porosimetry data for binder-free GT-MFI adsorbent pellets.

Property	Value
Real Bulk Density (g/cc)	0.945
Real Skeletal Density (g/cc)	2.150
Micropore Volume (cc/g) (N_2 physisorption)	0.180
Mesopore Volume (cc/g) (N_2 physisorption)	0.180
Macropore Volume (cc/g) (mercury porosimetry)	0.250

Table S9a. Estimate of the upper boundary for the different mass transfer coefficients at 323 K. Assume water as the solvent. ε_p = 0.43, d_p = 425 x 10^{-4} cm, $\dot{v}$ = 10 mL/min, D_c = 8 x 10^{-10} cm^2/s and r_c = 200 x 10^{-7} cm.

Component	k_{film} (min^{-1})	k_{pore} (min^{-1})	k_{micro} (min^{-1})	k_{app} (min^{-1})
**2-3,BDO**	70.3	1.77	1800	1.73
**Water**	135	4.69	1800	4.52
**Ethanol**	84.0	2.31	1800	2.25
**Maltose**	41.4	0.81	1800	0.79

Table S9b. Estimate of the lower boundary for the different mass transfer coefficients at 323 K. Assume water as the solvent. ε_p = 0.40, d_p = 600 x 10^{-4} cm, $\dot{v}$ = 1 mL/min, D_c = 1.0 x 10^{-11} cm^2/s and r_c = 300 x 10^{-7} cm.

Component	k_{film} (min^{-1})	k_{pore} (min^{-1})	k_{micro} (min^{-1})	k_{app} (min^{-1})
**2-3,BDO**	18.5	0.74	10.0	0.67
**Water**	35.4	1.96	10.0	1.57
**Ethanol**	22.1	0.97	10.0	0.85
**Maltose**	11.0	0.34	10.0	0.32

Table S9c. Estimated ranges for apparent mass transfer coefficient at 323 K. It should be noted that these values are not to be used as final parameters of the SMB and but instead as initial guesses and for regularization of the data fitting procedure.

Component	k_{app} (min^{-1})
**2,3-BDO**	0.67 — 1.73
**Water**	1.57 — 4.52
**Ethanol**	0.85 — 2.25
**Maltose**	0.32 — 0.79

Table S10. Parameters obtained from one iteration of the co-current method. Isotherm parameters were fitted to ternary 2,3-BDO/ethanol/water adsorption data (T = 296 K) using the MLL isotherm. This set of parameters was used as the initial guess to design the first SMB experiment.

Parameter	2,3-BDO	Water	Ethanol
q_m (g/cc_{MFI})	0.140	0.118	0.126
Affinity Constant K ($cc_{solution}$/g)	39.1	1.00	88.0
Henry's Constant H ($cc_{solution}$/cc_{MFI})	5.02x10^{-3}	3.48x10^{-2}	3.00x10^{-3}

Table S11. Breakthrough characteristics of 20 cm GT-MFI adsorption, and values of the isotherm and mass transfer parameters from fitting the model to measured data. Averages and standard deviations are obtained using each of the 8 columns individually. T = 323 K.

Mass of MFI in column (g)	8.7 ± 0.3
2,3-BDO/water selectivity	19 ± 5
2,3-BDO uptake (g/kg MFI)	88 ± 3
Column inner diameter (mm)	10
Column length (mm)	200
Column Bed Porosity, ε_b, (cm^3_{void}/cm^3_{bed})	0.344
Feed Flow Rate, q, (cm^3/min)	0.200
Peclet Number, Pe, (-) (Pe=uL/D_{ax})	250
Axial Dispersion, $D_{ax,}$ (cm^2/min)	0.076
Dead Volume, DV, (cm^3)	0.500
Particle Porosity, ε_p, (cm^3_{pore}/cm^3_{MFI})	0.405
$q_{m,BDO}$ (g/cc MFI)*	0.145 (0.140)
K_{BDO} (g/cc MFI)*	70.9 (38.4)
H_{BDO} (g/cc MFI)*	1x10^{-5} (6.57x10^{-5})
$k_{app,BDO}$ (g/cc MFI)*	0.962 (1.00)
$q_{m,Water}$ (g/cc MFI)*	0.132 (0.118)
K_{Water} (g/cc MFI)*	4.15 (1.00)
H_{water} (g/cc MFI)*	3.47x10^{-2} (2.97x10^{-2})
$k_{app,water}$ (g/cc MFI)*	1.49 (1.50)

* Fitted parameter (value in parenthesis denote initial guess)

Table S12. Evolution of the fitted parameters after each iteration k of the four small-scale SMB experiments. For instance, k=3 denotes the fitted parameters using data from experiments 1, 2 and 3. k=0 denotes the initial parameter guess, before any SMB experiment is conducted. Peclet number (Pe) = 250 and bed porosity (ε_b) = 0.335 for each column.

Parameters		k=0	k=1	k=2	k=3	k=4
**q_m (g/cc$_{MFI}$)**	**2,3-BDO**	0.140	0.140	0.140	0.107	0.107
	**Water**	0.118	0.118	0.129	0.200*	0.200*
	**Ethanol**	0.126	0.123	0.135	0.100**	0.100**
**K (cc/g)**	**2,3-BDO**	39.1	39.1	109	102	102
	**Water**	1.00	1.00	0.100**	1.21	1.21
	**Ethanol**	88.0	88.0	34.1	49.9	49.9
**H (cc/cc$_{MFI}$)**	**2,3-BDO**	$5.02x10^{-3}$	0.111	2.25×10^{-5}	1.24×10^{-3}	1.00×10^{-5}**
	**Water**	$3.48x10^{-2}$	9.70×10^{-2}	0.107	9.61×10^{-2}	9.17×10^{-2}
	**Ethanol**	$3.00x10^{-3}$	7.71×10^{-2}	5.75×10^{-3}	9.77×10^{-2}	9.64×10^{-2}
**k_{app} (min^{-1})**	**2,3-BDO**	0.962	0.65	1.18	0.679	0.659
	**Water**	1.50	1.48	0.918	0.893	0.877
	**Ethanol**	1.50	1.50	1.50	1.874	1.900*
	**ε_p (-)**	0.430	0.430	0.405	0.400**	0.400**
	**DV (cm^3/column)**	N/A	N/A	0.109	0.137	0.137

N/A: Dead volume (DV) was not included in the model.
*Fitted variable reached its upper boundary
**Fitted variable reached its lower boundary

Table S13. Lower and upper boundary limits for each parameter during the fitting procedure of the small-scale SMB experiments. The mass transfer bounds were based on the correlation results obtained in **Table S9**. For K and H, the bounds were determined based on the batch and breakthrough adsorption fitting results and are simply order of magnitude estimates. For the saturation capacity q_m, the upper bound is based on the micropore volume measured from N2 physisorption and the density of the adsorbate, which in practice represents the maximum selective adsorption capacity for each component. However, as was mentioned, some selective adsorption might occur in the small mesopore channels, and thus the upper bound of q_m was allowed to slightly exceed the adsorption in the micropore space. For BDO and ethanol, the lower q_m bound was a reasonable estimate based on the batch adsorption experiments and for water the lower boundary was allowed to be much smaller given the hydrophobic behavior of the MFI adsorbent.

**Parameters**		**Lower Bound**	**Upper Bound**
**q_m (g/cc$_{MFI}$)**	**2,3-BDO**	0.100	0.200
	**Water**	0.005	0.200
	**Ethanol**	0.100	0.200
**K (cc/g)**	**2,3-BDO**	10.0	150
	**Water**	1.00	5.00
	**Ethanol**	10.0	150
**H (cc/cc$_{MFI}$)**	**2,3-BDO**	$1.00\text{x}10^{-5}$	0.500
	**Water**	$1.00\text{x}10^{-5}$	0.500
	**Ethanol**	$1.00\text{x}10^{-5}$	0.500
**k_{app} (min^{-1})**	**2,3-BDO**	0.500	2.00
	**Water**	0.500	5.00
	**Ethanol**	0.500	3.00
	**ε_p (-)**	0.400	0.450
	**DV (cm^3/col)**	0.100	0.140

Table S14. Operating conditions of the four small-scale experiments.

Operating Variable	Experiment 1	Experiment 2	Experiment 3	Experiment 4
**Zone I Flow Rate (cm^3/min)**	2.43	2.83	2.92	1.58
**Zone II Flow Rate (cm^3/min)**	1.37	1.22	1.22	1.14
**Zone III Flow Rate (cm^3/min)**	1.78	1.50	2.20	1.50
**Zone IV Flow Rate (cm^3/min)**	1.15	1.15	1.17	1.06
**Step Time (t_{step}) (min)**	7.15	7.15	7.88	8.37

Table S15. Evolution of the root mean squared error (RMSE) for the four small-scale SMB experiments. RMSE computed for the concentration (g/L) of 2,3-BDO in the extract and raffinate and water in the extract after each iteration k.

RMSE (g/L)		k=1	k=2	k=3	k=4
**Extract**	**2,3-BDO**	2.83	1.01	0.382	5.09
	**Water**	$6.79x10^{-2}$	2.40	5.09	3.87
**Raffinate**	**2,3-BDO**	3.86	0.153	1.62	1.27

Table S16. Operating conditions for scaled-up SMB runs based upon the tuned model with parameters from **Table S11**. The input variables of our SMB system are: zone 1 flow rate, feed flow rate, extract flow rate, raffinate flow rate and step time.

Zone configuration	2-2-2-2
Zone 1 flow rate [ml/min]	12.93
Zone 2 flow rate [ml/min]	10.19
Zone 3 flow rate [ml/min]	12.63
Zone 4 flow rate [ml/min]	8.59
Feed flow rate [ml/min]	2.44
Extract flow rate [ml/min]	2.74
Desorbent flow rate [ml/min]	4.34
Raffinate flow rate [ml/min]	4.04
D/F ratio	1.77
Step time [min]	7.15
Column temperature [°C]	50
Adsorbent loading (g)	520

Table S17. Final set of isotherm and mass transfer parameters of all 14 components (ethanol + 13 components in the real broth feed).

Components	q_m (g/cc$_{MFI}$)	K (cc/g)	H (cc/cc$_{MFI}$)	k_{app} (1/min)
**BDO**	0.107	102.45	1.00x10^{-5}	0.659
**Water**	0.2	1.23	9.17x10^{-2}	0.877
**Ethanol**	0.1	49.9	9.64x10^{-2}	1.900
**Acetoin**	0.107	*65.0 (102.45)	1.00x10^{-5}	*0.520 (0.659)
**Maltose**	1.00x10^{-5}	1.00x10^{-5}	*0.115 (0.100)	0.877
**Xylose**	1.00x10^{-5}	1.00x10^{-5}	*0.150 (0.100)	0.877
**Arabinose**	1.00x10^{-5}	1.00x10^{-5}	*0.110 (0.100)	0.877
**Xylitol**	0.15	1.23	9.17x10^{-2}	0.877
**Glycerol**	0.15	*2.15 (1.23)	*0.15 (9.17x10^{-2})	*0.780 (0.877)
**Malic Acid**	0.15	1.23	*0.05 (9.17x10^{-2})	0.877
**Lactic Acid**	0.15	*1.00 (1.23)	*1.00 x10^{-3} (9.17x10^{-2})	*0.550 (0.877)
**Acetic Acid**	0.15	1.5	9.17x10^{-2}	*0.550 (0.877)
**Cl^-**	1.00x10^{-5}	1.00x10^{-5}	1.00x10^{-2}	1.00
**SO_4^{2-}**	1.00x10^{-5}	1.00x10^{-5}	1.00x10^{-5}	1.00

*Parameter was adjusted, value in parenthesis indicates initial guess

Table S18. Final set of the remaining SMB system parameters.

**Peclet Number (Pe)**	300
**Bed Porosity (ε_b)**	0.335
**Particle Porosity (ε_p)**	0.400
**Dead Volume DV (cm^3/col.)**	0.137

Table S19. Measured extract purity (ethanol-free basis) and recovery for all components in the real broth. Value in parenthesis indicate the prediction using the final model.

Components	Extract Purity (%)	Extract Recovery (%)
**BDO**	79.0 (77.3)	100 (96.9)
**Water**	19.6 (21.3)	3.06 (3.04)
**Acetoin**	0.40 (0.48)	75.8 (82.8)
**Maltose**	0.23 (0.29)	3.87 (4.42)
**Xylose**	0.07 (0.06)	3.48 (2.81)
**Arabinose**	0.14 (0.15)	4.01 (3.88)
**Xylitol**	0.07 (0.06)	3.51 (2.58)
**Glycerol**	0.22 (0.17)	4.26 (2.95)
**Malic Acid**	0.06 (0.06)	3.42 (2.79)
**Lactic Acid**	0.05 (0.05)	2.99 (2.42)
**Acetic Acid**	0.09 (0.09)	6.37 (5.74)
**Cl^-**	0.00 (0.005)	0.00 (1.03)
**SO_4^{2-}**	0.00 (0.00001)	0.00 (0.02)

Table S20. Breakthrough adsorption data for 30 cm columns. NB: no binder; B: binder.

Column	BDO uptake [g/kg MFI]	Separation Factor BDO/water
NB col1	79.5	29
B col1	79.2	17
NB col2	80.8	13
B col2	80.4	9
NB col3	79.7	42
B col3	78.5	26
NB col4	79.6	15
B col4	79.7	12
NB col5	75.6	18
B col5	76.2	92
NB col6	79.2	20
B col6	78.9	13
NB col7	77.7	36
B col7	75.0	8
NB col8	77.2	15
B col8	77.3	12

Table S21. Composition of cumulative extract product for SMB production run 1 after removing ethanol by vacuum distillation.

Components	Concentration in final product (g/L)
Maltose	2.7
Xylose	0.8
Arabinose	1.6
Xylitol	0.9
Glycerol	2.7
Malic acid	0.7
Lactic acid	0.8
Acetic acid	1.5
Acetoin	2.2
2,3-BDO	816.6
Water	9.8
Ethanol	97.0
Cl^-	0.0
SO_4^{2-}	0.0

Table S22. Feed concentration of the four small-scale SMB experiments.

Sample #	Experiment 1 Feed. Conc. (g/L)	Experiment 2 Feed. Conc. (g/L)	Experiment 3 Feed. Conc. (g/L)	Experiment 4 Feed. Conc. (g/L)
**2,3-BDO**[a]	93.6	95.2	94.3	94.2
**Water**[b]	898	921	929	933

[a]Measured with HPLC, [b]Measured with GC.

Table S23. Average extract concentration measurements for the four small-scale SMB experiments. For each sample, extract was collected for a full SMB cycle. Three different cycles were sampled for each experiment, all after cyclic steady state was achieved.

Component	Sample #	Experiment 1 Ex. Conc. (g/L)	Experiment 2 Ex. Conc. (g/L)	Experiment 3 Ex. Conc. (g/L)	Experiment 4 Ex. Conc. (g/L)
	**1**	38.9	16.2	42.1	85.8
	**2**	38.2	16.0	40.9	81.3
**2,3-BDO**[a]	**3**	37.2	16.2	40.7	83.6
	**1**	15.8	72.6	14.3	63.7
	**2**	15.7	72.3	14.1	61.2
**Water**[b]	**3**	15.6	72.8	13.7	62.6
	**1**	765	716	724	659
	**2**	742	719	729	673
**Ethanol**[b]	**3**	750	724	730	678

[a]Measured with HPLC, [b]Measured with GC.

Table S24. Average raffinate concentration measurements for the four small-scale SMB experiments. For each sample, raffinate was collected for a full SMB cycle. Three different cycles were sampled for each experiment, all after cyclic steady state was achieved.

Component	Sample #	Experiment 1 Raff. Conc. (g/L)	Experiment 2 Raff. Conc. (g/L)	Experiment 3 Raff. Conc. (g/L)	Experiment 4 Raff. Conc. (g/L)
	1	0.34	0	23.77	0
	2	0.38	0	24.15	0
2,3-BDO[a]	3	0.29	0	24.57	0
	1	559.85	267.41	836.35	684.09
	2	520.08	268.75	836.53	682.31
Water[b]	3	557.04	272.42	833.07	696.28
	1	379.4	580.54	144.93	283.62
	2	408	581.68	144.91	288.45
Ethanol[b]	3	379.02	587.89	146.17	295.91

[a]Measured with HPLC, [b]Measured with GC.

Table S25. Average recycle concentration measurements for the four small-scale SMB experiments. For each sample, the recycle stream was collected for a full SMB cycle. Three different cycles were sampled for each experiment, all after cyclic steady state was achieved.

Component	Sample #	Experiment 1 Recy. Conc. (g/L)	Experiment 2 Recy. Conc. (g/L)	Experiment 3 Recy. Conc. (g/L)	Experiment 4 Recy. Conc. (g/L)
	1	0	0	0	0
	2	0	0	0	0
2,3-BDO[a]	3	0	0	0	0
	1	0	0	0	0
	2	0	0	0	0
Water[b]	3	0	0	0	0
	1	778	778	778	778
	2	778	778	778	778
Ethanol[b]	3	778	778	778	778

[a]Measured with HPLC, [b]Measured with GC.

Table S26. Raw experimental data for batch adsorption experiments (296K)

Experiment Label[a]	m_{MFI} (g)	V_{in} (mL)	ρ_{in} (g/L)	ρ_{eq} (g/L)	$c_{in,BDO}$ (g/L)	$c_{in,water}$ (g/L)	$c_{in,EtOH}$ (g/L)
1-1	0.298	4.02	994	994	4.7	989	0
2-1	0.301	4.09	1000	1000	9.7	989	0
3-1	0.295	3.99	1000	1000	29.4	970	0
4-1	0.286	4.02	1000	1000	59.2	940	0
5-1	0.285	3.99	1000	1000	99.3	900	0
6-1	0.300	4.06	1000	1000	116.9	882	0
7-1	0.298	3.99	1000	1000	136	863	0
7-2	0.303	4.00	1006	1006	138.4	866	0
7-3	0.300	4.02	1000	1000	139.7	859	0
8-1	0.305	4.02	1000	1000	156.1	843	0
9-1	0.301	4.01	1000	1000	175	824	0
9-2	0.300	4.00	1000	1000	177.6	821	0
9-3	0.300	3.97	1009	1009	179.5	829	0
10-1	0.301	4.00	1000	1000	197.6	802	0
11-1	0.300	3.96	1013	1013	226.3	786	0
12-1	0.298	3.94	1022	1022	327.2	693	0
13-1	0.302	3.88	1030	1030	429.2	600	0
14-1	0.101	1.17	1002	1000	730.3	271	0
14-2	0.101	1.17	1006	1000	736	269	0
15-1	0.304	4.06	1000	1000	822.1	178	0
15-2	0.098	1.18	1001	1000	828.4	172	0
16-1	0.301	4.01	1000	1000	916	84	0
17-1	0.303	4.02	997	997	0	965	30.5
18-1	0.302	4.04	993	994	0	931	61.5
19-1	0.302	4.06	988	989	0	884	102.7
20-1	0.310	4.12	981	982	0	836	144.2
21-1	0.301	4.11	975	976	0	790	184
21-2	0.310	4.11	975	976	0	789	184.7
22-1	0.106	1.19	967	968	0	740	226.4
22-2	0.106	1.19	967	968	0	740	226.5
22-3	0.320	4.14	967	968	0	741	225.5
22-4	0.106	1.19	967	968	0	739	227.2
23-1	0.301	4.17	959	960	0	691	266.8
24-1	0.293	4.27	947	948	0	626	320.2
24-2	0.104	1.21	947	948	0	623	323.0
24-3	0.104	1.21	947	948	0	623	322.9
24-4	0.300	4.23	947	948	0	626	320.3
24-5	0.104	1.21	947	948	0	623	322.9
25-1	0.309	4.27	941	942	0	591	348.8

26-1	0.302	4.34	925	926	0	513	410.9
26-2	0.103	1.21	924	925	0	510	413.4
26-3	0.103	1.21	924	925	0	508	414.8
26-4	0.103	1.21	924	925	0	508	414.8
27-1	0.293	4.46	902	903	0	410	491.6
27-2	0.303	4.43	902	903	0	408	492.9
27-3	0.098	1.29	902	902	0	406	494.4
27-4	0.294	4.46	902	902	0	406	494.3
28-1	0.098	1.30	900	901	0	401	498.4
28-2	0.098	1.30	900	901	0	401	498.4
29-1	0.304	4.47	896	897	0	383	512.1
30-1	0.298	4.92	800	799	0	17	781.4
31-1	0.299	4.02	992	993	4.8	918	68.8
32-1	0.297	4.02	986	987	4.6	863	116.8
33-1	0.302	4.09	984	984	9.6	898	75.7
34-1	0.301	4.11	984	985	9.7	850	123.9
35-1	0.322	4.07	991	992	19.3	894	77.3
36-1	0.303	4.55	984	985	19.1	840	124.3
37-1	0.301	4.13	984	985	57.8	798	127.3
38-1	0.304	4.09	984	985	136.3	720	127.1
39-1	0.302	4.14	973	973	389.8	386	196.3
40-1	0.301	4.15	964	965	338.5	385	239.9
41-1	0.302	4.15	965	966	284.9	444	235.6

[a]The leading number in the label represents the experimental sample, while the trailing number represents the replicate (for instance for experiment sample 24 there were 5 replicates all with the same starting concentration).

Table S27. Equilibrium concentration (c_{eq}), volume (V_{eq}) and uptake (q_{eq}) based on batch adsorption experiments (296K). c_{eq} is a direct measurement while V_{eq} and q_{eq} are calculated using the pore-filling (PF) model described in the main manuscript. The average c_{eq} and q_{eq} values of each sample were used for fitting the MLL isotherm.

Experiment Label	$c_{eq,BDO}$ (g/L)	$c_{eq,water}$ (g/L)	$c_{eq,EtOH}$ (g/L)	V_{eq} (mL)	q_{BDO} (g/cc$_{MFI}$)	q_{water} (g/cc$_{MFI}$)	q_{EtOH} (g/cc$_{MFI}$)
1-1	3.60	991	0	3.97	15.5	164	0
2-1	7.40	993	0	4.03	32.6	147	0
3-1	24.8	975	0	3.94	66.7	113	0
4-1	53.4	947	0	3.97	91.1	88.8	0
5-1	93.3	907	0	3.93	101	78.8	0
6-1	111	889	0	4.00	101	78.2	0
7-1	130	870	0	3.94	109	70.9	0
7-2	132	873	0	3.94	104	75.9	0
7-3	133	867	0	3.96	114	66.1	0
8-1	150	851	0	3.96	114	65.7	0
9-1	170	830	0	3.95	98.4	81.7	0
9-2	172	828	0	3.95	108	71.9	0
9-3	173	836	0	3.91	112	68.2	0
10-1	192	808	0	3.95	109	70.7	0
11-1	220	793	0	3.91	121	58.4	0
12-1	321	701	0	3.89	145	35.0	0
13-1	424	605	0	3.83	137	42.7	0
14-1	728	272	0	1.15	136	43.8	0
14-2	730	270	0	1.15	154	25.3	0
15-1	822	178	0	4.01	145	34.7	0
15-2	827	173	0	1.16	151	29.1	0
16-1	918	81.8	0	3.96	136	44.1	0
17-1	0	973	24.1	3.97	0	67.6	88.9
18-1	0	939	54.7	3.99	0	53.2	100
19-1	0	893	95.9	4.01	0	43.7	107
20-1	0	845	138	4.07	0	38.2	112
21-1	0	799	177	4.06	0	23.6	124
21-2	0	798	178	4.05	0	31.8	117
22-1	0	749	220	1.17	0	34.9	114
22-2	0	749	220	1.17	0	33.6	116
22-3	0	748	220	4.09	0	41.6	110
22-4	0	749	220	1.17	0	24.6	123
23-1	0	699	261	4.12	0	26.2	122
24-1	0	633	316	4.22	0	25.3	123

24-2	0	633	316	1.19	0	3.93	139
24-3	0	633	316	1.19	0	7.77	136
24-4	0	632	316	4.17	0	35.1	115
24-5	0	633	316	1.19	0	7.27	137
25-1	0	597	345	4.21	0	29.2	119
26-1	0	519	407	4.28	0	23.1	124
26-2	0	516	409	1.19	0	27.2	120
26-3	0	516	409	1.19	0	9.21	134
26-4	0	516	409	1.19	0	9.5	134
27-1	0	414	489	4.41	0	26.8	121
27-2	0	411	491	4.38	0	37.8	112
27-3	0	410	492	1.28	0	32.0	117
27-4	0	410	492	4.41	0	29.7	119
28-1	0	407	495	1.28	0	5.92	137
28-2	0	407	495	1.28	0	6.03	137
29-1	0	388	509	4.42	0	16.3	129
30-1	0	17.5	782	4.86	0	13.2	132
31-1	4.30	926	62.3	3.97	7.43	48.7	97.6
32-1	4.40	872	110	3.97	3.44	38.8	109
33-1	8.70	906	69.6	4.04	13.5	48.0	93.5
34-1	9.30	859	118	4.05	7.01	38.7	106
35-1	17.8	904	70.6	4.02	21.9	35.6	96.5
36-1	18.4	849	118	4.50	13.6	29.5	108
37-1	56.4	806	122	4.08	28.7	31.4	94.6
38-1	134	728	123	4.04	52.4	25.5	80.1
39-1	391	388	194	4.08	55.1	40.6	66.4
40-1	338	390	237	4.10	63.8	11.3	82.2
41-1	284	448	233	4.10	55.9	28.7	75.2
42-1[a]	430	150	420	N/A	80.7	26.1	79.4

[a]Breakthrough experiment at 296K.

N/A: There is no V_{eq} for breakthrough experiments, c_{eq} is the concentration of the feed to the breakthrough column and q_{eq} was measured using the expressions found in the main manuscript for breakthrough experiments (maltose was used as the tracer).

S4. Supplementary Figures

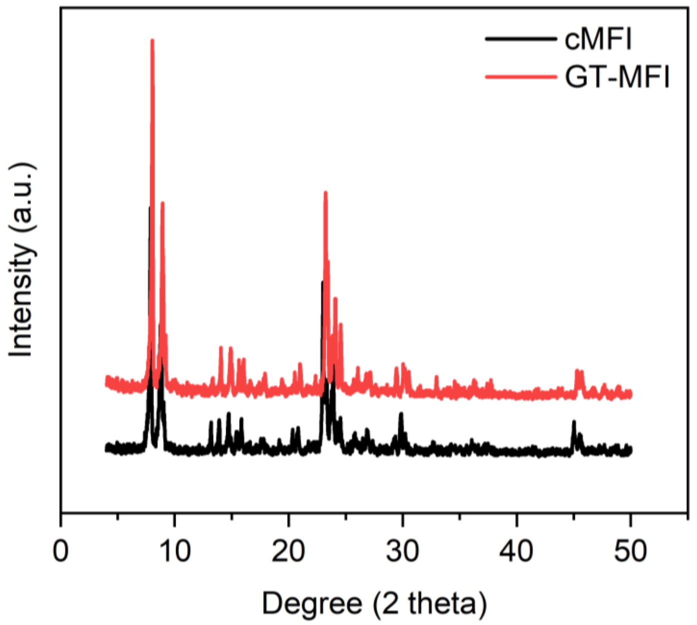

Figure S1. Powder XRD patterns of GT-MFI and cMFI zeolites.

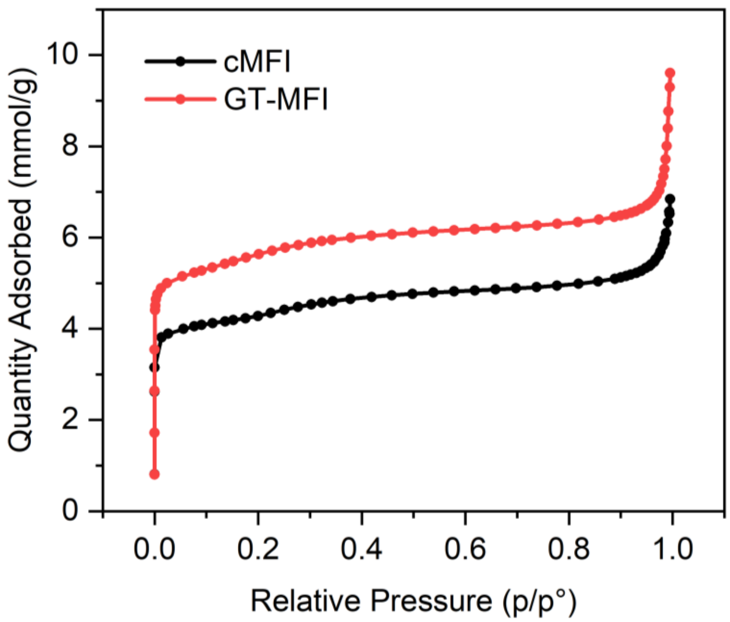

Figure S2. N_2 physisorption isotherms (P/P^0: $1.0*10^{-6}$ to 0.99) of GT-MFI and cMFI zeolites.

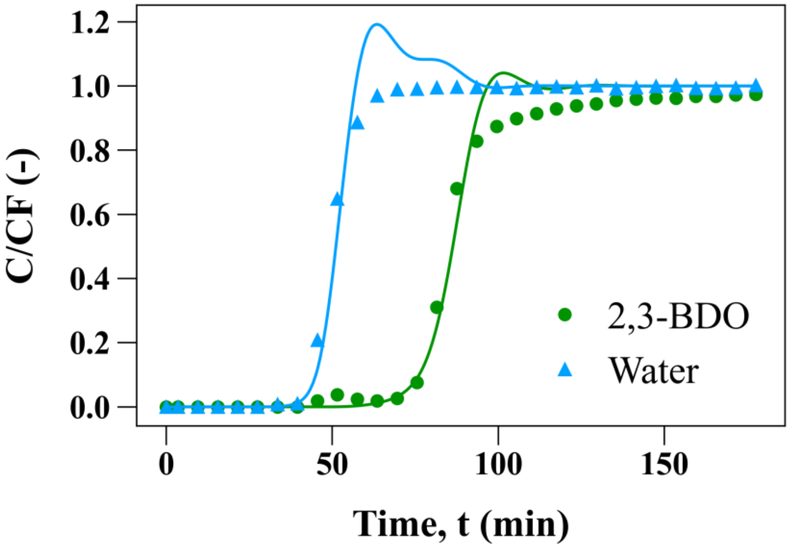

Figure S3. Breakthrough curve for 10 wt% 2,3-BDO in water (maltose was used as tracer). Symbols: experimental data, Solid curves: transport dispersive (TD) model fits.

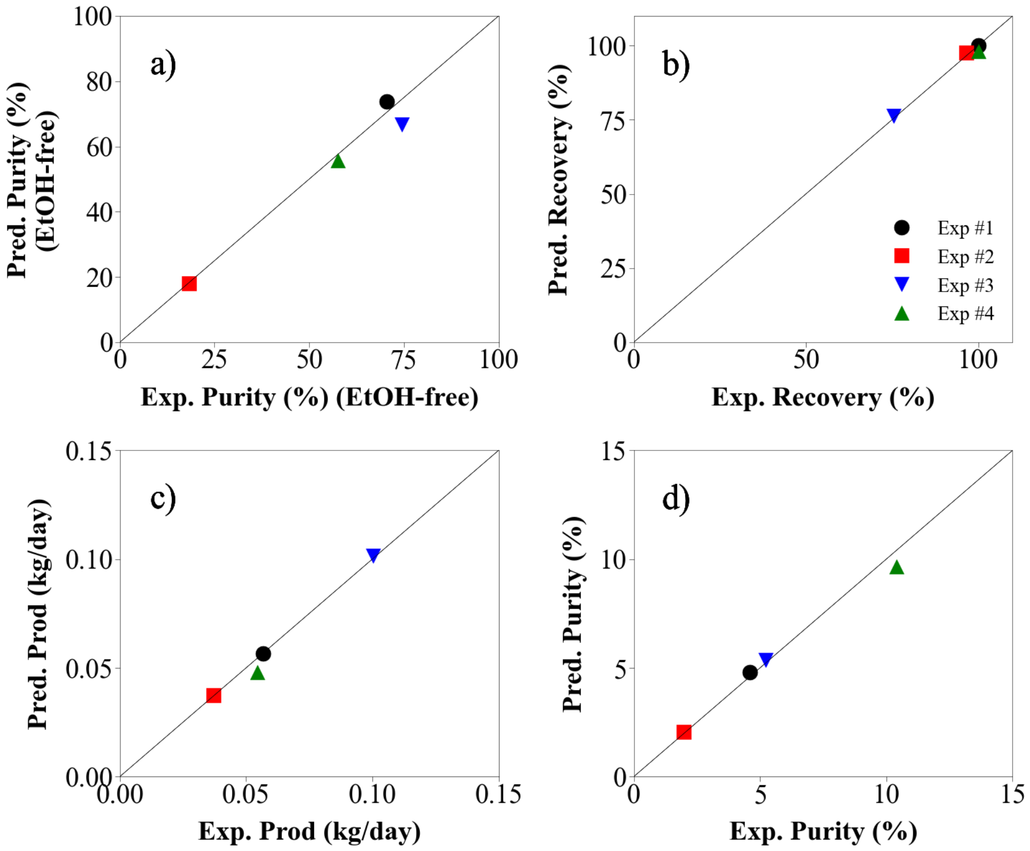

Figure S4. Parity plot showing the comparison between experimental and predicted performance metrics of the four SMB experiments at the small-scale. Performance metrics: (a) 2,3-BDO extract purity on an ethanol-free basis, (b) extract recovery, (c) productivity and (d) extract purity on an overall basis.

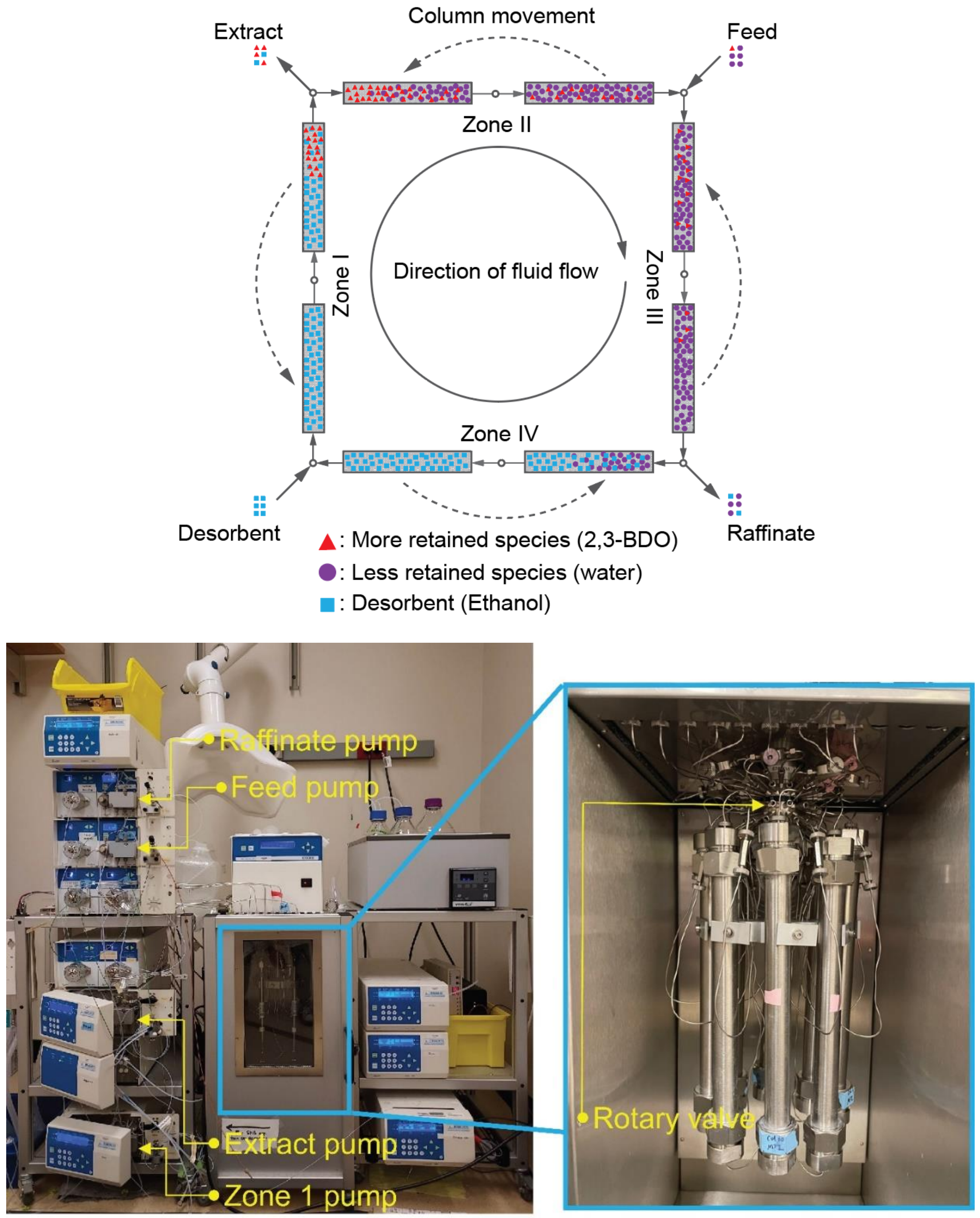

Figure S5. **Top**: schematic of a four-zone SMB (top) with 8 columns in a 2-2-2-2 configuration, with 2 inputs (feed and desorbent) and two outputs (extract and raffinate). **Bottom**: implementation of the 8-column SMB in a Knauer pilot unit. The columns are arranged in a circular format. Switching of the inlets and outlets is performed by physical rotation of the entire circular column setup.

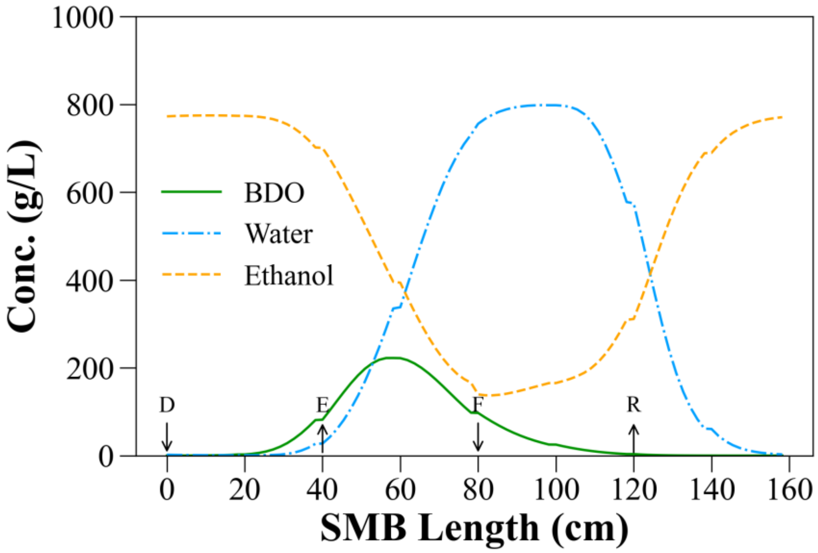

Figure S6. Predicted optimal SMB average internal concentration profile based. Isotherm and mass transfer parameters are based on the batch binary experiments and mass transfer correlations. This profile was used to select the concentrations of samples to be tested through batch adsorption experiments on 2,3-BDO/water/ethanol ternary mixtures. The objective function was productivity maximization subject to a minimum 2,3-BDO 75% extract purity (ethanol-free) and 95% recovery. To build this profile we used the isotherm parameters shown in **Table S7** and $k_{app,BDO} = 0.962$ min^{-1}, $k_{app,water} = k_{app,EtOH} = 1.50$ min^{-1} `

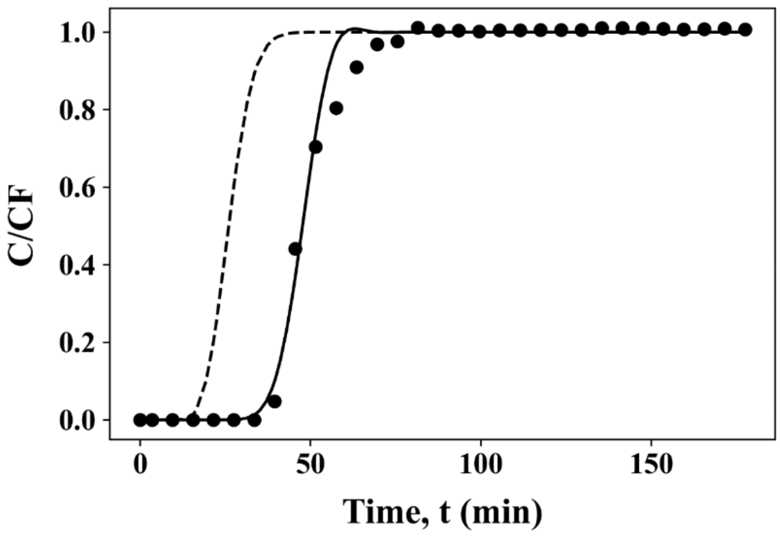

Figure S7. Comparison between experimental and predicted tracer breakthrough curve for maltose for the two proposed transport models (TDM and LDF). Peclet number (Pe) and bed porosity (ε_b) were fixed at 220 and 0.335, respectively, for both models. Fitted parameters for TDM were particle porosity (ε_p) and $k_{app,maltose}$, and their final values were 0.405 and 1.174 min^{-1}, respectively. No fitted parameters for LDF. Symbols: experimental, Solid Curve: fitted TDM model, Dashed Curve: fitted LDF model.

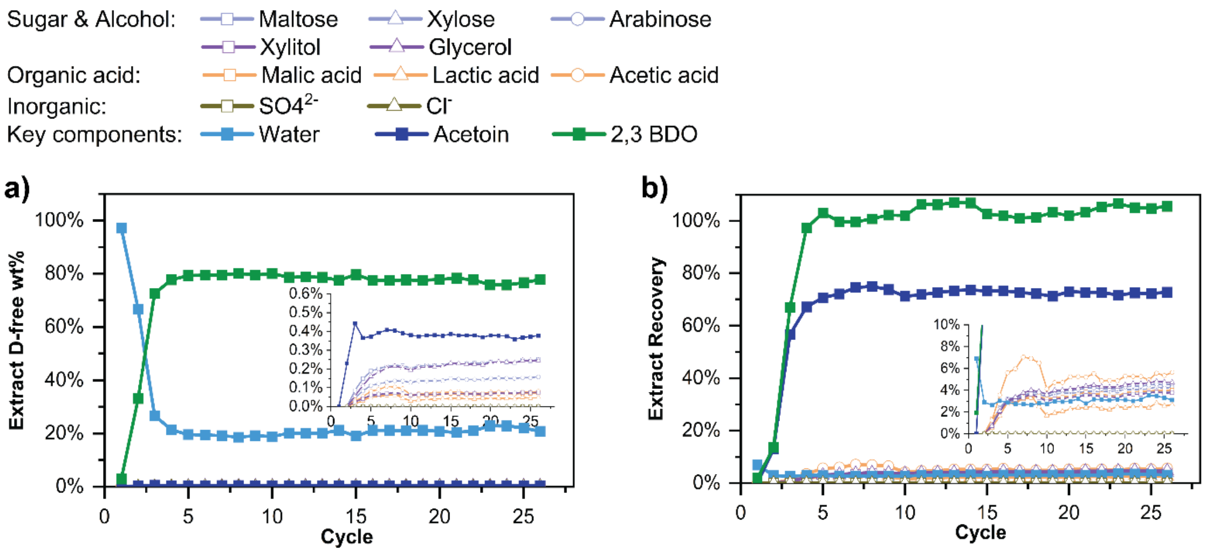

Figure S8. Transient evolution of the adsorption performance metrics for a repeated SMB production run (run 2) with the real fermentation broth: **(a)** Composition of the desorbent-free extract stream (inset shows the minor components in more detail), and **(b)** recovery of individual components in the extract. The total run time is 1487 min/26 cycles. The operation conditions is identical to the first production run.
